# Porcine Epidemic Diarrhea Virus Infection of Porcine Intestinal Epithelial Cells Causes Mitochondrial DNA Release and the Activation of the NLRP3 Inflammasome to Mediate Interleukin-1β Secretion

**DOI:** 10.3390/vetsci11120643

**Published:** 2024-12-12

**Authors:** Di Bao, Shushuai Yi, Luobing Zhao, Han Zhao, Jiuyuan Liu, Yiming Wei, Guixue Hu, Xinxin Liu

**Affiliations:** 1 College of Veterinary Medicine, Jilin Agricultural University, Changchun 130118, China; baodi666666@gmail.com (D.B.); zhaohan4566@163.com (H.Z.); liujiuyuan9916@163.com (J.L.); 18525182253@163.com (Y.W.); 2 College of Veterinary Medicune, Jilin Agricultural Science and Technology University, Jilin 132109, China; yss930117@163.com; 3 Institute of Animal Husbandry and Veterinary Medicine, Jilin Academy of Agricultural Sciences, Kemao Street No. 186, Gongzhuling 136100, China; a13630969537@163.com

**Keywords:** PEDV, mtDNA, NLRP3, IPEC-J2, IL-1β, inflammation

## Abstract

Porcine epidemic diarrhea virus (PEDV) induces enteritis and diarrhea in piglets. Mitochondrial DNA (mtDNA) contributes to virus-induced inflammatory responses; however, the involvement of inflammasomes in PEDV infection responses remains unclear. We investigated the mechanism underlying inflammasome-mediated interleukin (IL)-1β secretion during the PEDV infection of porcine intestinal epithelial (IPEC-J2) cells. IL-1β production and caspase-1 activity were assessed by quantitative PCR and enzyme-linked immunosorbent assay. NLRP3 inflammasome activation was assessed using immunoprecipitation experiments. Mitochondrial damage was evaluated by analyzing the mitochondrial membrane potential and ATP levels and by the flow cytometry examination of mitochondrial reactive oxygen species (mtROS). Mitochondria and mtDNA localization were observed using immunofluorescence. The inhibition of mtROS and mtDNA production allowed NLRP3 inflammasome and IL-1β expression detection and the evaluation of the pathway underlying NLRP3 inflammasome activation in PEDV-infected IPEC-J2 cells. IPEC-J2 cells upregulated IL-1β upon PEDV infection, where mature IL-1β secretion depended on caspase-1 activity, triggered NLRP3 inflammasome expression and assembly, and caused mitochondrial dysfunction, leading to mtDNA release and NLRP3 inflammasome activation, while mtROS contributed to NF-κB pathway activation, enhancing IL-1β secretion. This is the first demonstration of the mechanism underlying mtDNA release and NLRP3 inflammasome activation facilitating IL-1β secretion from PEDV-infected IPEC-J2 cells. These data enhance our understanding of the inflammatory mechanisms triggered by PEDV.

## Simple Summary

Porcine epidemic diarrhea virus (PEDV) poses a significant threat to piglets and severely undermines the healthy development of the pig breeding industry. This is primarily due to the fact that infection in piglets can lead to severe inflammatory diarrhea. The activation of inflammasomes within cells is closely linked to pathogen invasion and the stress imposed by the internal environment. This article aims to investigate the inflammatory response elicited by PEDV in cells and the potential mechanisms underlying the activation of inflammasomes. We conducted in vitro experiments to assess mitochondrial damage in cells following viral infection, measure the expression levels of the NLRP3 inflammasome and IL-1β, and evaluate the activation pathway of the NLRP3 inflammasome in PEDV-infected porcine small intestinal epithelial cells. Our findings indicate that PEDV induces mitochondrial dysfunction, facilitates the release of mitochondrial DNA, and activates the NLRP3 inflammasome. Concurrently, reactive oxygen species generated by mitochondria activate the NF-κB signaling pathway. Notably, inhibiting the release of mitochondrial reactive oxygen species and mitochondrial DNA reduces the IL-1β release associated with viral infection. This study underscores the potential of mitochondrial DNA as a target for the control of inflammation in cases of porcine epidemic diarrhea.

## 1. Introduction

Porcine epidemic diarrhea is an acute, highly contagious, intestinal infectious disease caused by porcine epidemic diarrhea virus (PEDV). PEDV infection can lead to acute viremia, severe atrophic enteritis (mainly in the jejunum and ileum), and increased inflammation and innate immune responses, with significantly elevated expression levels of pro-inflammatory cytokines, such as interleukin (IL)-1β, TNF-α, IL-6, and IL-8, having important roles in the PEDV infection process [1,2,3]. Inflammasomes are pattern recognition receptors in the cytoplasm with integral functions in innate immune responses [4]; they mainly comprise an inflammasome receptor (NLR or ALR), the adapter protein, ASC, and pro-caspase-1 [5]. When the inflammasome receptor is activated, ASC and pro-caspase-1 are recruited to form an inflammasome complex via the PYD and CARD domains [6]. The activated inflammasome induces the generation of caspase-1 protein with an effector function from pro-caspase-1, and active caspase-1 cleaves the IL-1β precursor, pro-IL-1β, to induce its maturation [7,8]. IL-1β secretion is regulated by two signaling pathways. The first is NF-κB signaling; after the activation of the NF-κB pathway, pro-IL-1β protein can be produced through nuclear transcription and translation [9,10,11]. In the second signaling pathway regulating IL-1β secretion, pathogen-associated molecular patterns or damage-associated molecular patterns (DAMPs) activate the inflammasome by inducing specific physiological changes in cells, leading to pro-IL-1β cleavage by caspase-1 and the secretion of mature IL-1β to exert its biological functions [12]. Various viruses, including severe acute respiratory syndrome coronavirus 2 (SARS-CoV-2), human immunodeficiency virus (HIV), hantavirus, African swine fever virus, pseudorabies virus, porcine reproductive and respiratory syndrome virus (PRRSV), and Newcastle disease virus, can induce the maturation and secretion of IL-1β/IL-18, leading to pyroptosis through the activation of the inflammasome assembly, and thereby enhance the inflammatory response [13]. The infection of porcine intestinal epithelial cells (IPEC-J2) by porcine epidemic diarrhea virus (PEDV) can activate NF-κB signaling through Toll-like receptors (TLRs) 2, 3, and 9. Notably, TLR2 plays a significant role in the activation of NF-κB through the PEDV structural N protein [14]. Simultaneously, the non-structural PEDV protein, nsp4, also activates NF-κB signaling to upregulate IL-1α, IL-1β, and TNF-α and inhibit PEDV replication [1]. Transcriptome analysis showed that IL-1β was significantly upregulated in the PEDV-infected small intestinal mucosa [15]; however, the detailed molecular mechanism by which the inflammasome mediates IL-1β production after PEDV infection has yet to be clarified.

Mitochondria are the main source of ATP and are important in fighting viral infections [16,17]. Mitochondria can release substances, such as reactive oxygen species (ROS) and mitochondrial DNA (mtDNA), in response to cellular stress and the loss of homeostasis [18]. These substances are commonly known as DAMPs, and their release triggers an immune response. Dengue virus (DENV) and PRRSV can both induce mtDNA release and induce ROS production, as well as mediating inflammasome activation [19,20]. Further, the influenza A virus M2 protein can trigger mtDNA release in the cytoplasm and stimulate cGAS- and DDX41-dependent innate immune responses [21]. To date, most investigations of PEDV-mediated mitochondrial antiviral signals have focused on apoptosis and found that PEDV 3CLpro can cause the collapse of the mitochondrial membrane potential [22]; however, the inflammatory mechanism mediated by PEDV remains unclear. 

In this study, we first demonstrated that IL-1β was secreted following the PEDV infection of IPEC-J2 cells and then investigated whether IL-1β secretion during the infection process was mediated by the NLRP3 inflammasome. In addition, we explored whether mtDNA release after PEDV infection mediated mitochondrial dysfunction and whether this was related to NLRP3 inflammasome activation. Our study is the first to assess the inflammatory mechanism by which PEDV activates the NLRP3 inflammasome through mtDNA release to mediate IL-1β secretion. Our findings provide a useful reference for the study of the inflammatory mechanisms involved during PEDV infection and provide new insights to inform the development of targeted drugs.

## 2. Materials and Methods

### 2.1. Cell Lines and Virus Strains

The porcine intestinal epithelial cell line IPEC-J2 and PEDV strain CV777 were maintained by the School of Veterinary Medicine, Jilin Agricultural University. IPEC-J2 cells were recovered and cultured in DMEM F12 (SH30271.FS, HyClone, Logan, UT, USA) containing 10% fetal calf serum (04-001-1ACS, BI, Kibbutz Beit Hamek, Israel). Cells were digested with 0.25% trypsin (SH30042.01, HyClone, Logan, UT, USA) for passage.

After the IPEC-J2 cells reached 80% confluence, the culture medium was discarded under sterile conditions, the cells were rinsed three times with sterile PBS, and the virus solution was then added. Then, the cells were placed in a 37 °C constant-temperature incubator for 1–2 h to allow the virus to bind to the cell membrane. Next, the inoculum was discarded, the cells were washed three times with PBS, and serum-free DMEM F12 was added, before incubation in a 37 °C constant-temperature incubator to wait for cell lesions to develop.

### 2.2. Quantitative PCR

Total cellular RNA was extracted using a total cell RNA extraction kit (BSC60S1, Bioer, Hangzhou, China) and then reverse-transcribed for fluorescence quantitative PCR experiments. The primers used to amplify the porcine *IL-1β* (M86725.1), *NLRP3* (JQ219660.1), and *ASC* (AB873106.1) sequences published in the GenBank database by real-time PCR were designed using the Primer Premier 5.0 software. The *GAPDH* gene was used as an internal control. The primers were synthesized by Shanghai Sangon Bioengineering Co., Ltd. (Shanghai, China), and their sequences are listed in Table 1.

### 2.3. siRNA

Lipofectamine 3000 transfection reagent (L3000001, Invitrogen, Waltham, MA, USA) was used according to the manufacturer’s instructions. The reagent was mixed with p3000 and siRNA and incubated for 15 min before being added to a six-well plate. The NLRP3 siRNAs used for transfection were synthesized by Saisuofei Biotechnology Co., Ltd. (Wuxi, China), and their sequences are provided in Table 2.

### 2.4. Enzyme-Linked Immunosorbent Assay (ELISA)

The porcine IL-1β levels were assessed using an ELISA kit (MB-2562A, Enzyme Biotechnology, Shanghai, China). Briefly, 100 μL cell supernatant samples were added to ELISA reaction plates and incubated at room temperature for 2 h; then, the samples were aspirated and the plates washed five times. Next, a detection antibody was added and the samples incubated at room temperature for 1 h before the antibody was discarded and the plates were washed five times. Then, 100 µL enzyme working reagent was added to each well, the plates were incubated at room temperature for 30 min, the solution was discarded, and the samples were washed seven times. A one-step substrate reagent was added to each well and they were incubated for 30 min at room temperature. Then, 50 µL stop solution was added to each well, and the absorbance at 450 nm was read within 15 min. Finally, a standard curve was drawn to calculate the IL-1β protein content in the cell supernatant samples.

### 2.5. Caspase-1 Activity Detection

The cell culture medium was discarded and the cells washed three times with PBS, followed by the addition of 100 µL of cell lysis solution. A caspase-1 activity detection kit (C1102, Beyotime, Shanghai, China) was used to set up reactions, which were incubated at 37 °C for 1–2 h. Then, the absorbance was measured on a microplate reader at 405 nm, a standard curve drawn, and the caspase-1 enzyme activity calculated.

### 2.6. Western Blot Analysis

The PEDV nucleocapsid, N, IL-1β, NLRP3, ASC, caspase-1, p65, and pp65 protein levels were analyzed by Western blotting. Specifically, the cell supernatants were discarded and the cells were washed twice with PBS and then lysed in lysis buffer (PC101, Yazyme, Shanghai, China) containing protease inhibitors. The protein concentration was determined using a BCA kit (A55860, Thermo, Waltham, MA, USA) and the protein concentrations balanced by dilution with lysis buffer. After the separation of the protein samples by 10–12% SDS polyacrylamide gel electrophoresis, the samples were transferred to a polyvinylidene fluoride membrane (IPVH00010, Millipore, Darmstadt, Germany), which was then blocked with a quick blocking solution (PS108P, Yazyme, Shanghai, China) for 15 min and incubated with the primary antibody overnight. The membranes were washed five times and then incubated with HRP-labeled anti-rabbit secondary antibody (S0001, Affinity, Jiangshu, China) for 60 min. After washing the membrane a further five times, the Western blots were observed using a chemiluminescence imaging system (GS-800; Bio-Rad, Shanghai, China).

The primary antibodies used in this study included mouse monoclonal PEDV N (BGT-ANT-37707, Biogradetech, Waltham, MA, USA), rabbit polyclonal anti-IL-1β (bs-0812R, Bioss, Beijing, China), rabbit monoclonal ASC (WL02462, Wanleibio, Shenyang, China), rabbit monoclonal NLRP3 (WL02635, Wanleibio, Shenyang, China), rabbit monoclonal IL-1β P17 (WL00891, Wanleibio, Shenyang, China), rabbit polyclonal antibody caspase-1 (22915-1-AP, Proteintech, Chicago, IL, USA), rabbit monoclonal NF-κB p65 (AF5006, Affinity, Jiangshu, China), NF-κB pp65 (AF2006, Affinity, Jiangshu, China), and rabbit monoclonal GAPDH (GB15004-100, Servicebio, Wuhan, China).

### 2.7. Immunoprecipitation Assay

IPEC-J2 cells were stimulated with 100 ng/mL lipopolysaccharides (LPS; L2630, Sigma, St. Louis, MA, USA) for 9 h; then, ATP (HY-B2176, MCE, Monmouth, NJ, USA) was added at a final concentration of 5 mmol for 4 h to generate the LPS+ATP stimulation group. Simultaneously, cells to be tested were collected for lysis according to the method described above (Section 2.6). NLRP3 or ASC antibodies (5 µg) were mixed with the cleaved protein, and the primary antibody homologous serum was used as a control. Then, the samples were incubated overnight at 4 °C to form antigen–antibody complexes. The magnetic beads in the classic Protein A/G immunoprecipitation kit (YJ201, Yazyme, Shanghai, China) were washed, added to the antigen–antibody complex, mixed well, and incubated at 4 °C overnight. The beads were then collected using a magnetic stand, the supernatant discarded, and the beads washed three times with PBS. Finally, the proteins bound to the beads were eluted for Western blot analysis.

### 2.8. Inhibitor Treatment of IPEC-J2 Cells

The tool at https://www.medchemexpress.cn/dilution-calculator.htm (accessed on 10 October 2024) was used to calculate the specific concentration of each inhibitor, which was then added to the cell culture medium and processed for 1 h, followed by the replacement of the culture medium. The inhibitors used included Mito-TEMPO (HY-112879, MCE, Monmouth, NJ, USA), to inhibit mitochondrial ROS production; Ac-YVAD-cmk (HY-16990, MCE, Monmouth, NJ, USA), to inhibit caspase-1 activity; BAY11-7082 (HY-13453 MCE, Monmouth, NJ, USA), to inhibit NF-κB pathway activation; and MCC950 (HY-12815, MCE, Monmouth, NJ, USA), to inhibit NLRP3 inflammasome activation.

### 2.9. Detection of Mitochondrial Membrane Potential, ATP, and Mitochondrial Reactive Oxygen Species (mtROS)

The mitochondrial membrane potential was detected by staining cells with JC-1 dye (C2006, Beyotime, Shanghai, China) for 30 min and observing the fluorescence intensity of JC-1 monomers (green fluorescence) and JC-1 aggregates (red fluorescence) using a fluorescence microscope (BX53, Olympus, Tokyo, Japan). After the IPEC-J2 cells were treated with the lysis buffer from the enhanced ATP detection kit (S0027, Beyotime, Shanghai, China), they were centrifuged at 12,000× *g* for 5 min and the supernatants collected to analyze the total ATP levels. To detect the mtROS levels, a MitoSOX Red mitochondrial superoxide probe (40778ES50, Yeasen, Shanghai, China) was used to detect the fluorescence intensity of the ROS produced by the mitochondria in the cells. The probe was diluted to a concentration of 1 µM with PBS. After the cells were washed, the probe was added and the samples incubated in a 37 °C water bath for 30 min in the dark, followed by the analysis of the fluorescence levels using a flow cytometer.

### 2.10. mtDNA Localization

The IPEC-J2 cells were washed three times with HBSS, Mito-Tracker Red CMXRos (C1049B, Beyotime, Shanghai, China) was used to label mitochondria, and an appropriate amount of probe was added, according to the reagent instructions, and it was incubated with the cells for 30 min. After washing the cells three times with HBSS, an appropriate amount of culture medium was added and the cells incubated with Picogreen (12641ES01, Yeasen, Shanghai, China) for 1.5 h, followed by the observation of colocalization under a fluorescence microscope.

### 2.11. DNase I Protein Transfection

Cells were grown to 80% confluence in 24-well culture plates before protein transfection. To prepare the protein transfection complex, 5 μg DNase I (HY-108882, MCE, Monmouth, NJ, USA) and Starvio (T81001, EMI Biotechnology, Changzhou, China) were mixed, according to the instructions. After standing for 3 min, the Trans buffer was added to 50 μL and incubated for 15 min. During this period, the cells were washed twice with 37 °C preheated PBS and then 500 μL serum-free DMEM F2 added. Next, 50 μL of the protein transfection complex was added to the serum-free DMEM F2, and the samples were placed in a 37 °C constant-temperature cell incubator and incubated for 4 h to complete protein transfection.

### 2.12. Statistical Analyses

Data are expressed as the mean and standard deviation. The *t* test was used to compare the mean values between two groups, and a one-way analysis of variance with the Bonferroni correction was applied to compare the mean values among multiple groups. Data were analyzed using the GraphPad Prism v8.0 software. Significance was defined as *p* < 0.05.

## 3. Results

### 3.1. PEDV Infects IPEC-J2 Cells to Induce IL-1β Production

**Figure 1:**
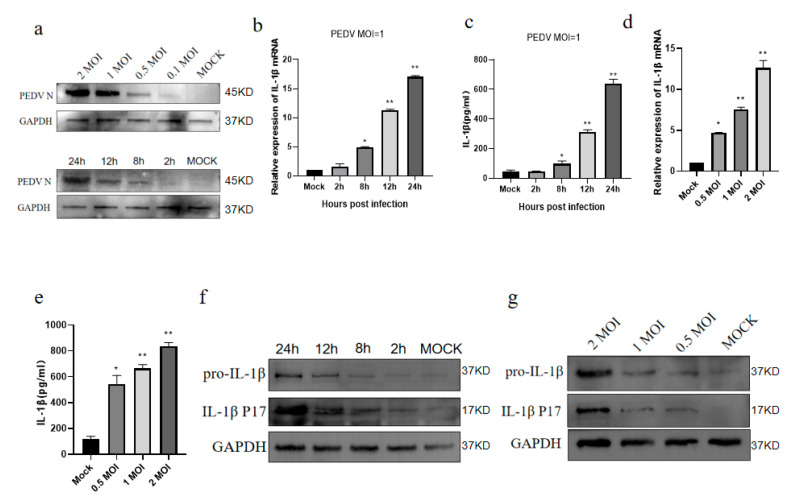
Porcine epidemic diarrhea virus (PEDV) infects IPEC-J2 cells to promote the secretion of mature interleukin (IL)-1β. PEDV (multiplicity of infection (MOI) = 1) was used to infect IPEC-J2 cells for the specified periods of time. Detection of PEDV N protein expression via Western blot following PEDV infection in IPEC-J2 cells (**a**). mRNA expression (**b**) and Western blot (**f**) analyses of IL-1β levels, as well as the results of an enzyme-linked immunosorbent assay (ELISA) to assess IL-1β secretion in the cell supernatants (**c**). IPEC-J2 cells were infected with PEDV at the specified dose for 24 h, followed by fluorescence quantification (**d**), Western blotting (**g**), and the ELISA detection of IL-1β secretion in the cell supernatant (**e**). Data represent the mean ± SD (*n* = 3). * *p* < 0.05, ** *p* < 0.01.

IL-1β is an inflammation-related cytokine that also has key roles in cell proliferation, differentiation, and apoptosis [23,24]. PEDV infection increases the expression of pro-inflammatory cytokines and chemokines in in vitro experiments, and pro-inflammatory cytokines, such as IL-1β, inhibit PEDV replication in vitro [14,25]. Therefore, we first assessed the IL-1β expression levels in IPEC-J2 cells after PEDV infection. The Western blot analysis was conducted on IPEC-J2 cells infected with PEDV for 24 h. The results demonstrated that the N protein of PEDV increased in a dose-dependent manner. Furthermore, the level of the N protein in IPEC-J2 cells rose with the extension of the infection duration. These findings suggest that PEDV replicates efficiently in IPEC-J2 cells (Figure 1a). The real-time quantitative PCR analysis demonstrated that the *IL-1β* mRNA expression levels showed an upward trend 8 h after the infection of the IPEC-J2 cells with PEDV and reached a peak at 24 h (Figure 1b). Further, the ELISA of the cell supernatants revealed that the IL-1β secretion from PEDV-infected IPEC-J2 cells also showed a comparable upward trend (Figure 1c). In addition, infection with different doses of PEDV led to a dose-dependent increasing trend in *IL-1β* mRNA expression (Figure 1d), while the IL-1β secretion in the cell supernatants also increased in a dose-dependent manner, according to the ELISA results (Figure 1e). The Western blot analysis demonstrated an increasing trend in the IL-1β protein levels with an increasing PEDV exposure time and dose. During this period, the mature IL-1β P17 protein also showed the same increasing trend (Figure 1f,g). Taken together, these results indicate that the PEDV infection of IPEC-J2 cells promotes IL-1β expression and secretion.

### 3.2. PEDV Infection of IPEC-J2 Cells Induces Increased Caspase-1 Enzyme Activity

**Figure 2:**
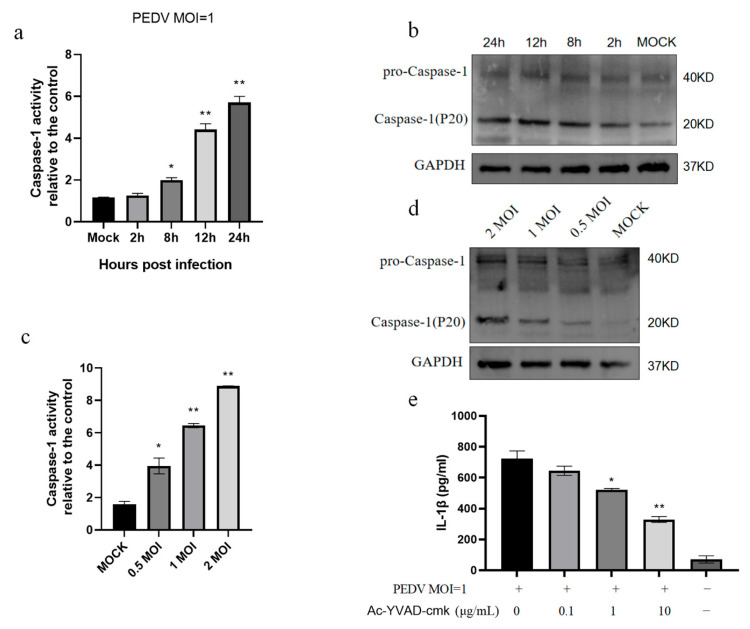
PEDV promotes caspase-1 enzymatic activity in IPEC-J2 cells. Caspase-1 enzyme activity (**a**) and Western blot results (**b**) at the specified time points after the PEDV infection of IPEC-J2 cells (MOI = 1). Caspase-1 enzyme activity (**c**) and Western blot results (**d**) of IPEC-J2 cells infected with PEDV at the specified doses for 24 h. IPEC-J2 cells were treated with the caspase-1 inhibitor, Ac-YVAD-cmk, at the specified concentration for 1 h and then inoculated with PEDV (MOI = 1). ELISA was used to detect IL-1β secretion in the cell supernatant 24 h after infection (**e**). Data represent the mean ± SD (*n* = 3). * *p* < 0.05, ** *p* < 0.01.

Enzymatically active caspase-1 protein is necessary for the secretion of biologically active IL-1β [26]. Therefore, we analyzed the enzymatic activity of caspase-1 after the PEDV infection of IPEC-J2 cells and found that the caspase-1 enzyme activity increased (Figure 2a). Further, the Western blot assays demonstrated that the P20 protein expression increased with the infection time after cleavage by caspase-1 (Figure 2b). Following the infection of IPEC-J2 cells with different doses of PEDV, the caspase-1 enzyme activity increased in a dose-dependent manner (Figure 2c,d). In addition, the treatment of IPEC-J2 cells with the caspase-1 inhibitor, Ac-YVAD-cmk, reduced IL-1β secretion (Figure 2e). Together, these data indicate that the PEDV infection of IPEC-J2 cells promotes caspase-1 enzymatic activity and that IL-1β secretion depends on this caspase-1 activity.

### 3.3. PEDV-Infected IPEC-J2 Cells Activate the NLRP3 Inflammasome

**Figure 3:**
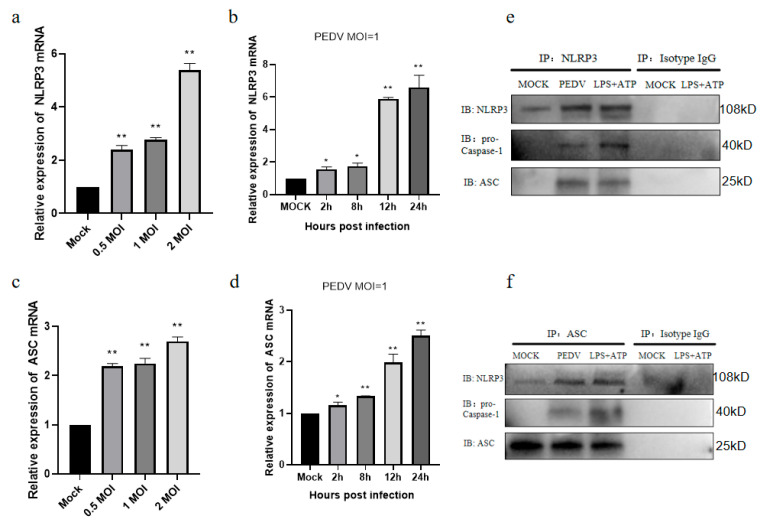
PEDV infection of IPEC-J2 cells activates the NLRP3 inflammasome. PEDV was used to infect IPEC-J2 cells at the specified doses for 24 h and then the *NLRP3* inflammasome (**a**) and *ASC* (**c**) mRNA expression were analyzed. *NLRP3* (**b**) and *ASC* (**d**) mRNA expression in IPEC-J2 cells infected with PEDV (MOI = 1) for the specified times. NLRP3 (**e**) and ASC (**f**) rabbit-derived primary antibodies were used as bait antibodies and rabbit serum was used as the negative control antibody in a co-immunoprecipitation experiment. Data represent the mean ± SD (*n* = 3). * *p* < 0.05, ** *p* < 0.01.

Our findings demonstrate that the PEDV infection of IPEC-J2 cells promotes the secretion of mature IL-1β. Previous studies have shown that RNA viruses can induce mature IL-1β secretion by activating the NLRP3 inflammasome pathway at different stages of infection [27,28,29]. To determine whether the NLRP3 inflammasome is activated after PEDV infection, we detected its expression and found that the *NLRP3* inflammasome mRNA expression increased in a time- and dose-dependent manner following the infection of IPEC-J2 cells with PEDV (Figure 3a,b), demonstrating that the PEDV infection of IPEC-J2 cells promoted *NLRP3* mRNA expression. Further, the *ASC* mRNA expression was also upregulated after the PEDV infection of IPEC-J2 cells (Figure 3c,d). Next, a co-immunoprecipitation experiment using A/G magnetic beads was conducted, with the NLRP3 and ASC antibodies as bait and primary antibody homologous serum as a negative control. The results showed that IPEC-J2 cells infected with PEDV and cells in the LPS+ATP induction group contained NLRP3 assembled in inflammasomes with ASC and pro-caspase-1 (Figure 3e,f). In summary, the NLRP3 inflammasome was activated in PEDV-infected IPEC-J2 cells.

### 3.4. PEDV-Infected IPEC-J2 Cells Induce IL-1β Production by Activating the NLRP3 Inflammasome

**Figure 4:**
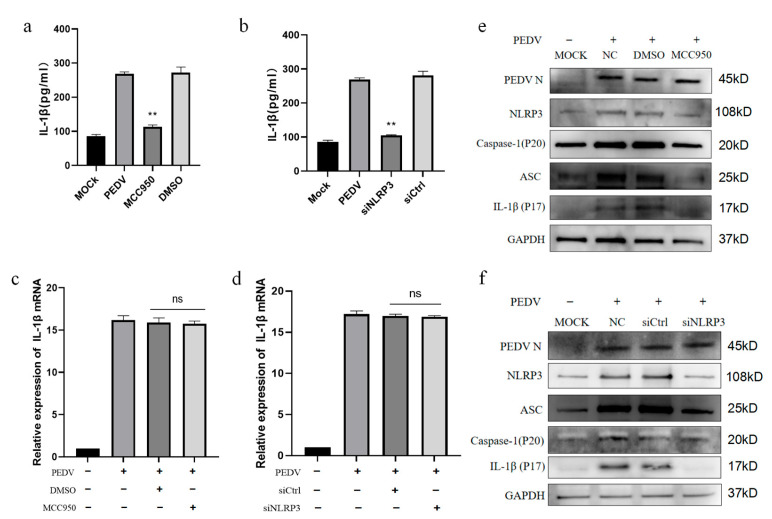
PEDV-infected IPEC-J2 cells secrete IL-1β through NLRP3 inflammasome activity. IPEC-J2 cells were treated with 10 µM MCC950 for 1 h, and negative control cells were treated with DMSO for the same time. After 24 h of cell infection with PEDV (MOI = 1), IL-1β secretion in the cell supernatants was detected by ELISA (**a**) and *IL-1β* mRNA quantified by fluorescence (**c**). siNLRP3 and siCtrl (control) were transfected into IPEC-J2 cells. PEDV was used to infect cells (MOI = 1) for 24 h, IL-1β secretion in the cell supernatants was detected by ELISA (**b**), and *IL-1β* mRNA expression was quantified by fluorescence (**d**). Western blot of IPEC-J2 cells treated with 10 µM MCC950 (**e**) and siNLRP3 transfection of IPEC-J2 cells to detect the expression of inflammasome proteins (**f**). Data represent the mean ± SD (*n* = 3),** *p* < 0.01.

Next, we determined whether IL-1β production induced by the PEDV infection of IPEC-J2 cells was related to NLRP3 inflammasome activation. Cells were treated with the NLRP3 inhibitor MCC950 and the amount of IL-1β secreted in the cell supernatants was detected after PEDV infection. The results showed that, after NLRP3 inflammasome inhibition, IL-1β secretion in the cell supernatants was significantly decreased (Figure 4a). In addition, siRNA transfection was used to silence the *NLRP3* inflammasome mRNA, which also resulted in significantly reduced IL-1β secretion in the cell supernatant (Figure 4b); however, neither MCC950 nor siNLRP3 significantly influenced *IL-1β* mRNA transcription in IPEC-J2 cells (Figure 4c,d). Nevertheless, the Western blot analysis of the inflammasome protein components showed that treatment with MCC950 and SiNLRP3 inhibited the expression of ASC, caspase-1, and the IL-1β p17 protein in the cells (Figure 4e,f). Together, these results indicate that PEDV-infected IPEC-J2 cells induce IL-1β secretion by activating the NLRP3 inflammasome.

### 3.5. PEDV Infection of IPEC-J2 Cells Causes Mitochondrial Dysfunction, Leading to mtROS Production and mtDNA Release

**Figure 5:**
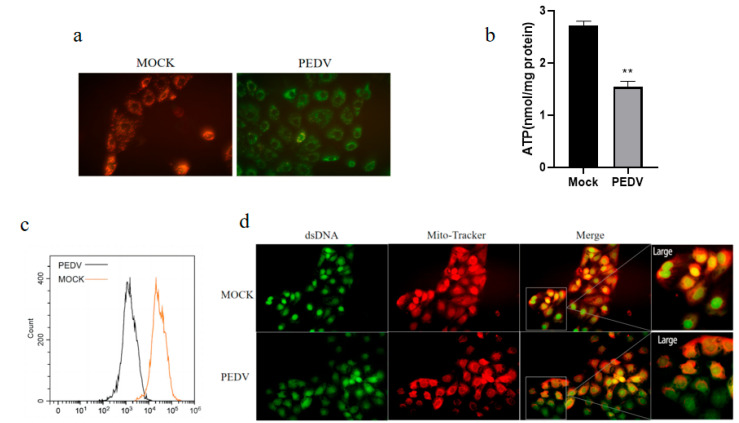
PEDV infection causes mitochondrial dysfunction and results in mitochondrial reactive oxygen species (mtROS) production and mitochondrial DNA (mtDNA) release. Mitochondrial membrane potential was decreased (**a**). Reduced ATP production after PEDV infection (**b**). Flow cytometry fluorescence intensity analysis of mtROS production after PEDV infection (**c**). Immunofluorescence showing mtDNA release after PEDV infection (**d**). Data represent mean ± SD (*n* = 3), ** *p* < 0.01.

Mitochondria participate in a wide range of innate immune pathways, and there is increasing evidence supporting a role for mitochondria in promoting innate immune activation after cell injury and stress through ROS production [16,30], where mtROS can accelerate the activation of the NLRP3 inflammasome [31]. In addition, various physiological or pathological stressors can also trigger mtDNA release into the cytoplasm, thereby activating the NLRP3 inflammasome [32,33]. To clarify the role of mitochondria in mediating inflammasome activity in PEDV-infected IPEC-J2 cells, we studied the mitochondrial function of IPCJ2 cells during PEDV infection. The mitochondrial membrane potential was detected by JC-1 fluorescent molecule staining after PEDV infection. We observed that PEDV infection reduced the mitochondrial membrane potential (Figure 5a). In addition, PEDV infection significantly reduced the ATP production by IPEC-J2 cells (Figure 5b). These findings show that PEDV infection causes mitochondrial dysfunction. Next, we used MitoROS probes to selectively target mitochondria in living cells; MitoROS probes are rapidly oxidized by superoxide and cannot be oxidized by other ROS or reactive nitrogen species. Oxidation products emit strong fluorescence in cells; therefore, we stained the ROS produced by the mitochondria after PEDV infection and analyzed them through flow cytometry. The results showed that PEDV infection caused mtROS accumulation in the cells (Figure 5c). In addition, we stained IPEC-J2 cells infected with PEDV with Mitotracker (red, mitochondrial indicator) and Picogreen (green, mitochondrial DNA and nuclear DNA indicator). Subsequent immunofluorescence microscopy observation demonstrated that mtDNA and mitochondria were increased in non-overlapping staining areas after PEDV infection, indicating that part of the mtDNA was released outside of the mitochondria (Figure 5d). These data indicate that the PEDV infection of IPEC-J2 cells can lead to mitochondrial dysfunction, the accumulation of mtROS, and the release of mtDNA.

### 3.6. mtROS Activates NF-κB Signaling After PEDV Infection of IPEC-J2 Cells

**Figure 6:**
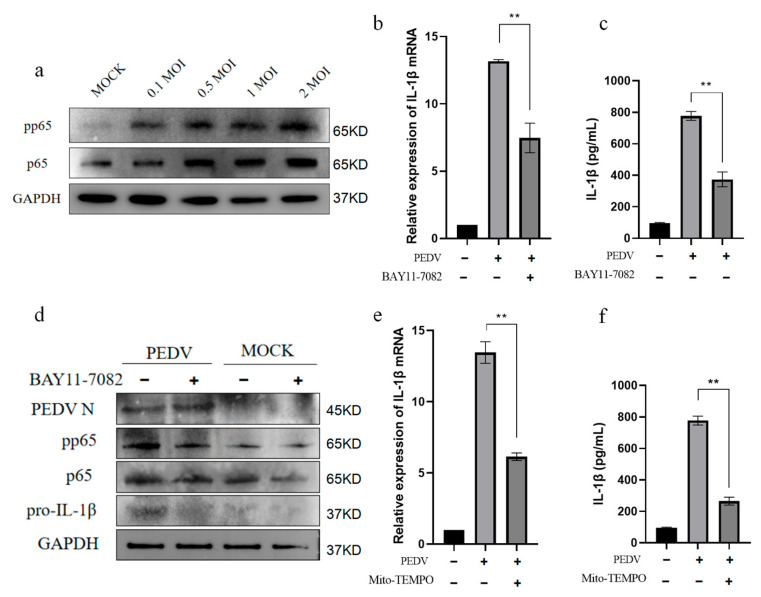
mtROS are involved in NF-κB activation in PEDV-infected IPEC-J2 cells. IPEC-J2 cells were infected with PEDV at the specified doses for 24 h and the expression levels of pp65 and p65 detected by Western blot (**a**). IPEC-J2 cells were treated with the NF-κB inhibitor BAY11-7082 (10 μM). *IL-1β* mRNA expression was detected 24 h after PEDV infection (MOI = 1) (**b**) and IL-1β secretion in the cell supernatant detected by ELISA (**c**). Western blot to assess the expression of pp65, p65, and IL-1β proteins (**d**). After IPEC-J2 cells were treated with 10 µM Mito-TEMPO for 1 h, *IL-1β* mRNA expression was detected following PEDV infection (MOI = 1) for 24 h (**e**) and IL-1β secretion in the cell supernatant detected by ELISA (**f**). Data represent mean ± SD (*n* = 3), ** *p* < 0.01.

NF-κB is a nuclear transcription factor with an important role in the regulation of inflammatory responses. PEDV can activate NF-κB through the TLR2, TLR3, and TLR9 pathways [14], and some studies have confirmed that ROS can also activate the NF-κB pathway to regulate *IL-1β* mRNA transcription [34,35]. Our data demonstrate that mitochondrial stress can lead to mtROS accumulation in the cytoplasm after the PEDV infection of IPEC-J2 cells. Based on this finding, we speculated that mtROS production after PEDV infection may be involved in NF-κB activation. To test this hypothesis, we explored the role of mtROS in inducing IL-1β production in IPEC-J2 cells infected with PEDV. Western blot experiments showed that P65 and pp65 were upregulated in a dose-dependent manner after PEDV infection (Figure 6a), indicating that NF-κB was activated. Further, the NF-κB inhibitor, BAY11-7082, significantly inhibited PEDV infection-mediated IL-1β mRNA expression and protein secretion (Figure 6b,c). Furthermore, Western blotting showed that BAY11-7082 alleviated pp65 phosphorylation and reduced pro-IL-1β protein expression (Figure 6d). Treating IPEC-J2 cells with the mitochondrial superoxide-targeted inhibitor Mito-TEMPO significantly inhibited *IL-1β* mRNA production mediated by the PEDV infection of IPEC-J2 cells (Figure 6e,f). These results indicate that mtROS are involved in NF-κB signaling activation after the PEDV infection of IPEC-J2 cells.

### 3.7. PEDV-Infected IPEC-J2 Cells Activate the NLRP3 Inflammasome Through mtDNA Release

**Figure 7:**
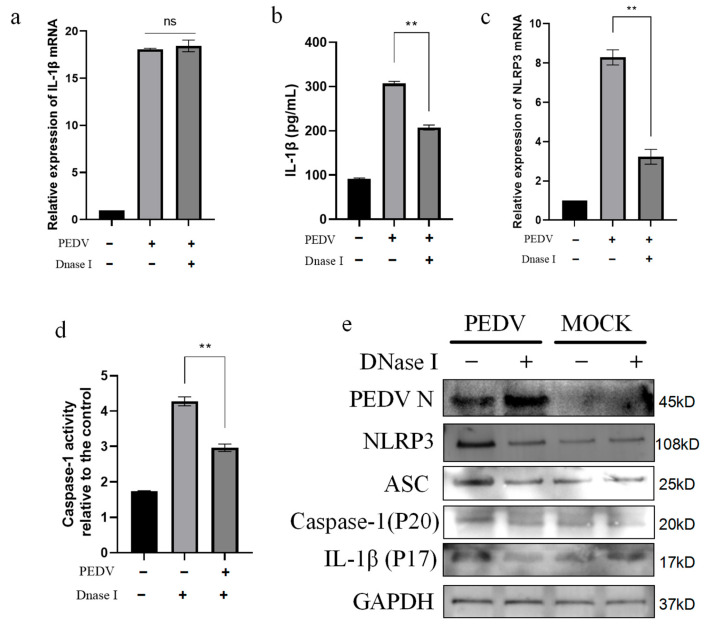
mtDNA participates in NLRP3 inflammasome activation in PEDV-infected IPEC-J2 cells. After transfection with DNase I, IPEC-J2 cells were infected with PEDV (MOI = 1). IL-1β mRNA expression (**a**) and secretion in cell supernatants detected by ELISA (**b**). *NLRP3* inflammasome mRNA expression (**c**) and caspase-1 enzyme activity (**d**). Western blot detection of NLRP3 inflammasome and downstream protein expression after transfection with DNase I protein (**e**). Data represent mean ± SD (*n* = 3). ** *p* < 0.01.

Oxidative stress in mitochondria is an important reason for mtDNA release [36]. Further, mtDNA is a recognized DAMP, and mitochondrial oxidative stress can cause mtDNA to translocate into the cytoplasm and activate the NLRP3 inflammasome [33,37]. To determine whether the mtDNA produced by mitochondrial stress oxidation after the PEDV infection of IPEC-J2 cells is involved in the NLRP3 inflammasome activation process, we next inhibited mtDNA synthesis using DNase I and observed the NLRP3 activation after PEDV infection. The results showed that *IL-1β* mRNA transcription was not affected by DNase I transfection (Figure 7a); however, it significantly inhibited IL-1β secretion into the cell supernatant (Figure 7b). In addition, after DNase I transfection, the *NLRP3* inflammasome mRNA expression was reduced in the cells (Figure 7c), while the caspase-1 enzyme activity decreased (Figure 7d). The Western blot analysis also showed that the protein expression levels of NLRP3, ASC, caspase-1, and IL-1β were inhibited after DNase I transfection (Figure 7e). These results indicate that mtDNA activates the NLRP3 inflammasome in PEDV-infected IPEC-J2 cells.

## 4. Discussion

Since the coronavirus disease 2019 (COVID-19) pandemic, the inflammatory pathogenic mechanisms of coronaviruses have become an important focus of widespread interest worldwide. The new coronavirus mediates IL-1β secretion by activating the NLRP3 inflammasome, leading to a systemic inflammatory storm [38]. The porcine enteric coronavirus, transmissible gastroenteritis virus, can also mediate pyroptosis in host cells by activating the NLRP3 inflammasome [27]; however, the pathway mediating inflammasome activation during PEDV-induced inflammatory responses has not been completely elucidated. PEDV infection can cause acute enteritis and fatal watery diarrhea in piglets [15]. KEGG pathway analysis showed that the inflammatory bowel disease pathway was highly enriched after the co-infection of PK15 cells with PEDV and BVDV [39]. Therefore, understanding the detailed molecular mechanisms underlying inflammatory responses may contribute to the prevention and treatment of PEDV infection.

IL-1β is a pyrogen and an endogenous cell mediator. Numerous studies have proven that PEDV infection can promote inflammatory cytokine secretion and lead to intestinal inflammation [15,25]. A transcriptome analysis of IPEC-J2 cells infected with PEDV found that the expression of IFN-α, IFN-β, TNF-α, IL-6, IL-8, and IL-12 was increased 12 h after infection, while their levels were not significantly affected during the early stage of infection [40]. In this study, we found that PEDV significantly promoted IL-1β secretion during 12 h of IPEC-J2 cell infection and that the levels peaked at 24 h (Figure 1a), indicating that the process of PEDV’s induction of inflammatory factor expression is related to the temporal progression of PEDV infection. Therefore, we speculate that PEDV may inhibit inflammation by producing certain proteins required for its own replication in the early stages of infection and activate innate immune responses in the later stages of infection. This phenomenon is also reflected in the process of SARS-CoV-2 infection, in which the non-structural proteins 1 and 13 can inhibit the NLRP3 inflammasome activation process to meet the early replication needs of the virus [41]. Inflammation generation is a double-edged sword: appropriate inflammation induction will activate downstream mechanisms, thereby inhibiting viral replication, while excessive activation will have adverse effects on the host [42]. Viruses can regulate inflammation to satisfy their own replication needs; hence, inflammation induction may be related to the PEDV replication mechanism.

IPEC-J2 cells are porcine intestinal cells that primarily replicate in the small intestines of pigs following infection with PEDV, and they exhibit high sensitivity to the virus [43,44]; consequently, IPEC-J2 cells were selected as the model for this study. Previous research has demonstrated that the PEDV infection of Vero cells can induce pyroptosis via the NLRP3 inflammasome pathway [45], a finding that aligns with the expression trend of the NLRP3 inflammasome observed in IPEC-J2 cells in the present study. Conversely, some studies have indicated that the expression of inflammatory factors is downregulated following PEDV infection. For example, Liu et al. reported that the levels of the NLRP3 inflammasome, p65, and pp65 were downregulated in porcine alveolar macrophages post-infection with PEDV [46]. This suggests that PEDV may exert an inhibitory effect on the cellular innate immune inflammatory pathway. Variations in the results across different cell types may be attributable to factors including immune tolerance and viral virulence.

During IL-1β production, NF-κB regulates *IL-1β* transcription, while the inflammasome mediates IL-1β secretion [47,48]. Therefore, we studied the specific process involved in the secretion of the inflammatory cytokine IL-1β after the PEDV infection of IPEC-J2 cells, and we found that viral infection activated the NLRP3 inflammasome in this context (Figure 3). Although we detected an interaction between ASC and the NLRP3 inflammasome protein in the MOCK group using the ASC bait antibody, our results confirm that PEDV infection enhanced the binding of the NLRP3 inflammasome, ASC, and pro-caspase-1 (Figure 3e,f). After we inhibited the NLRP3 inflammasome, both IL-1β secretion (Figure 4a,b) and caspase-1 (P20) protein expression (Figure 4e,f) were reduced. These results indicate that PEDV-infected IPEC-J2 cells secrete IL-1β through the NLRP3 inflammasome pathway. Numerous studies have demonstrated that, after its secretion, IL-1β can activate intracellular NF-KB signaling through IL-1R, to induce positive self-transcriptional regulation [49,50,51]. We found that the NF-KB signaling pathway was activated after PEDV infection (Figure 6a), and we speculate that, after PEDV infects IPEC-J2 cells, it may also mediate NF-KB signaling pathway activation through the IL-1β paracrine pathway, thus aggravating the inflammatory process. Further, NF-KB signaling activation can mediate myosin light chain kinase secretion and affect tight junction proteins, resulting in increased intestinal permeability and diarrhea [52,53]. We speculate that the PEDV infection process may be related to the above pathways. The detailed mechanisms underlying PEDV-mediated inflammatory diarrhea warrant further study.

Mitochondria are not only the main source of ATP but also participate in many other cell signaling events, such as the regulation of Ca^2+^ homeostasis, apoptosis, and ROS production [54]. mtROS production is essentially a physiological response to resist pathogenic invasion, which is beneficial in inhibiting the viral infection process. mtROS clearance increases the viral titer in nasal epithelial cells upon infection with influenza A virus [55]. Further, mtROS can amplify mitochondrial antiviral signaling and RIG-I-like receptor-mediated antiviral responses [56]; however, excessive mtROS production can mediate inflammatory reactions and cause physiological damage [57]. This has also been confirmed during SARS-CoV-2 infection. The mtROS content produced by infected human alveolar epithelial cells is markedly increased compared with extracellular ROS production, prolonging oxidative stress and exacerbating inflammation [58]. We detected mtROS production after the PEDV infection of IPEC-J2 cells by flow cytometry and found that mtROS were also highly accumulated in the cytoplasm of IPEC-J2 cells (Figure 5c). Further, treating the cells with mtROS inhibitors significantly inhibited IL-1β mRNA transcription and protein secretion in the cell supernatants (Figure 6e). This finding indicates that mtROS may act as an agonist to increase IL-1β production during IL-1β generation by PEDV-infected IPEC-J2 cells.

mtDNA is present in mitochondria and, after release, acts as a DAMP, thereby mediating cellular inflammatory responses [59]. Many viral infections can induce host cells to produce immune responses through mtDNA; for example, HIV can exert inflammatory effects by increasing plasma mtDNA [60]. Further, DENV can induce mtDNA release in the cytoplasm, upregulate TLR9, and influence downstream signaling events [19], while PRRSV activates the NLRP3 inflammasome through cytoplasmic mtDNA stress [20]. Interestingly, we also observed similar results in PEDV-infected IPEC-J2 cells. By detecting the localization of mitochondria and mtDNA, we found that mtDNA was released into the cytoplasm after PEDV infection (Figure 5d) and activated the NLRP3 inflammasome in IPEC-J2 cells (Figure 7e). mtDNA release is related to mitochondrial dysfunction, and the released mtDNA can directly bind to pattern recognition receptors and inflammasomes to enhance pro-inflammatory responses [61].

## 5. Conclusions

**Figure 8:**
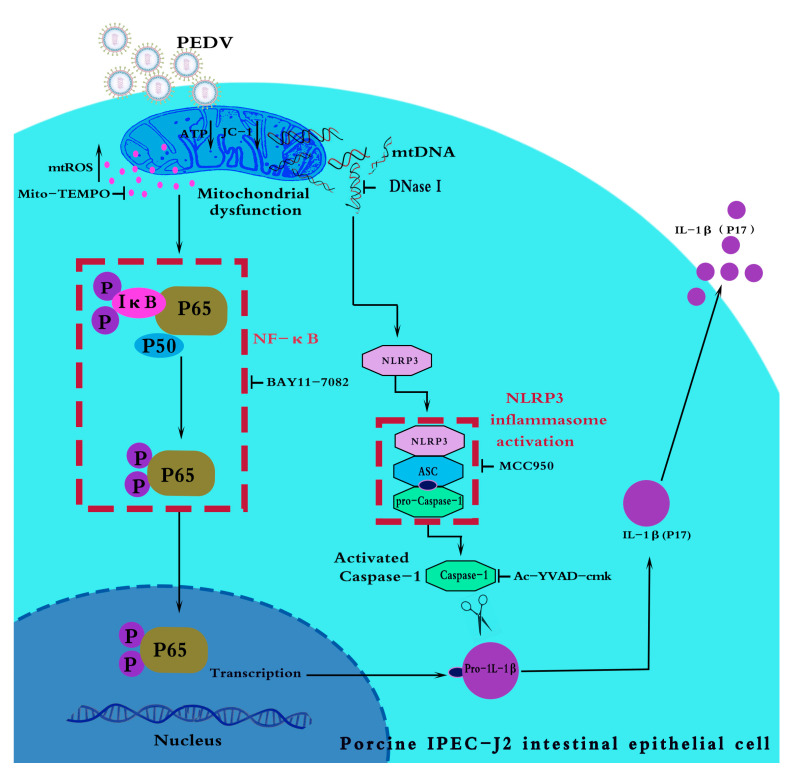
Schematic showing that PEDV infection leads to cytoplasmic mitochondrial DNA release and the activation of the NLPR3 inflammasome.

In conclusion, we observed that the PEDV infection of IPEC-J2 cells caused mitochondrial dysfunction, mtROS production, and mtDNA release, to activate the NF-κB signaling pathway and NLRP3 inflammasome, thereby inducing IL-1β production. We report, for the first time, that the NLRP3 inflammasome is activated after the PEDV infection of IPEC-J2 cells, mediating IL-1β secretion. Our data also indicate that mitochondria are critical in PEDV-triggered inflammatory responses (Figure 8). The results of this research enrich our understanding of the inflammatory mechanisms occurring during PEDV infection and provide new ideas to inform the development of anti-PEDV-targeted drugs.

## Figures and Tables

**Table 1:** qPCR primer sequences.

Gene	Forward Primer (5′–3′)	Reverse Primer (5′–3′)
IL-1β	AAGAGGGACATGGAGAAGCGATTTG	TTGTTCTGCTTGAGAGGTGCTGATG
NLRP3	TGTATTGAGAACTGTCGCCATGTGG	CTCCTCTTCCTCCTCCTCCTCTTTG
GAPDH	GATTCCACCCACGGCAAGTTCC	AGCACCAGCATCACCCCATTTG
ASC	GAAGGTGCTGACGGAAGAGC	TCCTTGCAGGTCAGGTTCCA

**Table 2:** Synthetic siRNA sequences.

siRNA	Sense (5′–3′)	Anti-Sense (5′–3′)
siNLRP3	CGGUUAAGUUGUCUCAAAUTT	AUUUCACAGTTCUUAAGGCTT
siCtrl	UUCUCCGAACGUGUCACGUTT	ACGUGACACGUUCGGAGAATT

## Data Availability

The original contributions presented in the study are included in the article/Supplementary Materials, further inquiries can be directed to the corresponding author.

## Supplementary Materials

The following supporting information can be downloaded at: https://www.mdpi.com/article/10.3390/vetsci11120643/s1. The original Western blots will be published as Supplementary Materials.
