# Supplementary material for: Porcine Epidemic Diarrhea Virus Infection of Porcine Intestinal Epithelial Cells Causes Mitochondrial DNA Release and the Activation of the NLRP3 Inflammasome to Mediate Interleukin-1β Secretion
Source: Vet Sci. 2024 Dec 12;11(12):643. doi: 10.3390/vetsci11120643 (PMC11680147; doi:10.3390/vetsci11120643)
Supplement: Supplementary file 1 [file vetsci-11-00643-s001.zip › Supplementary Materials/Western blot.pdf]

Supplementary Materials

Fig 1 a

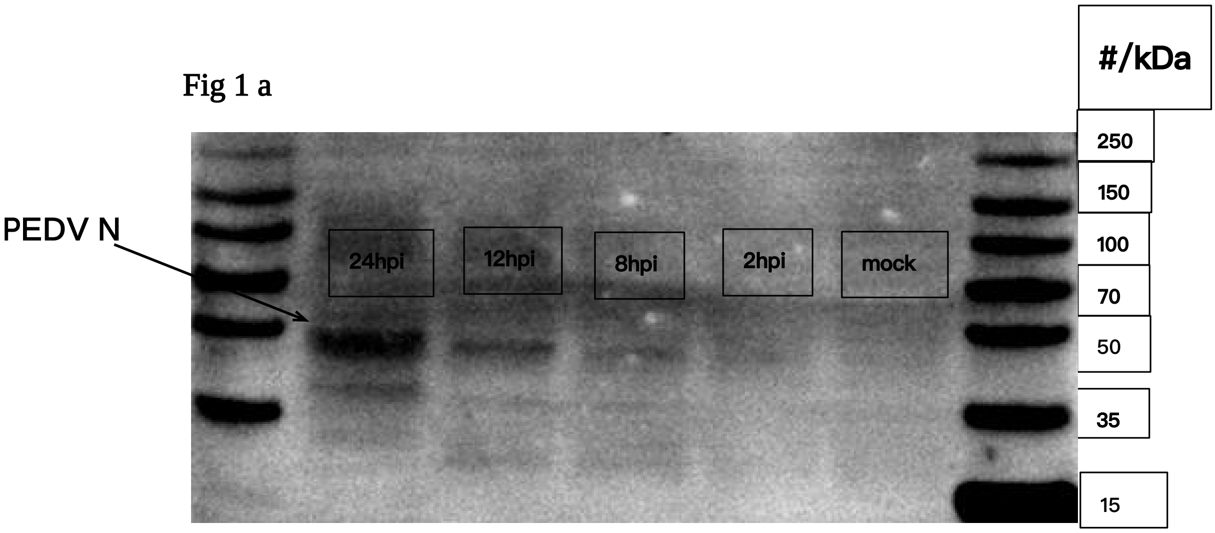

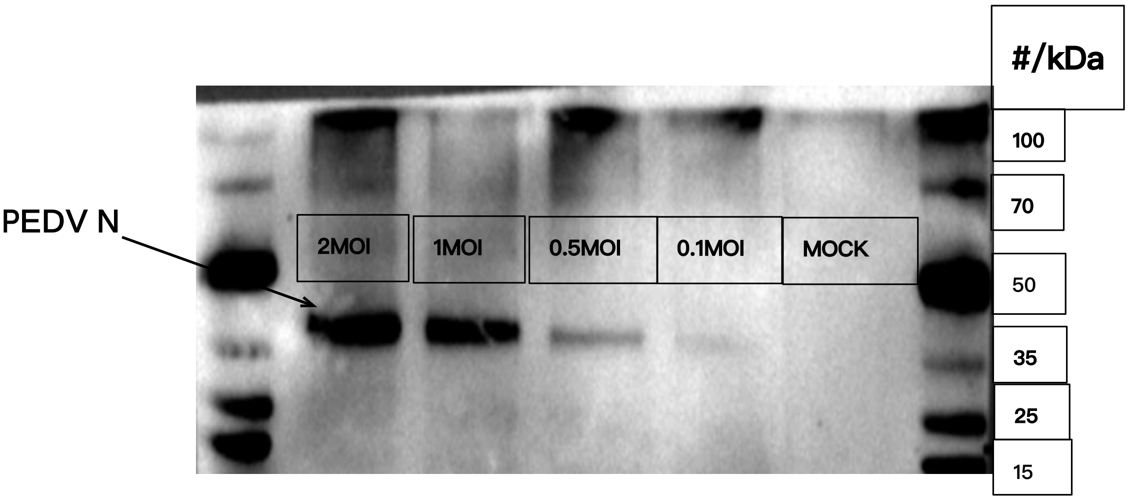

Fig 1a

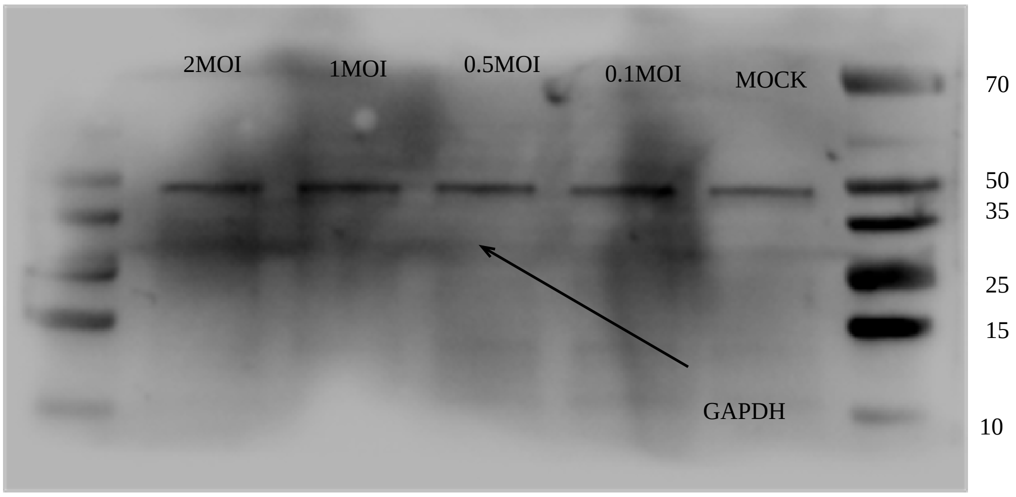
2MOI
1MOI
0.5MOI
0.1MOI
MOCK
70
50
35
25
15
GAPDH
10

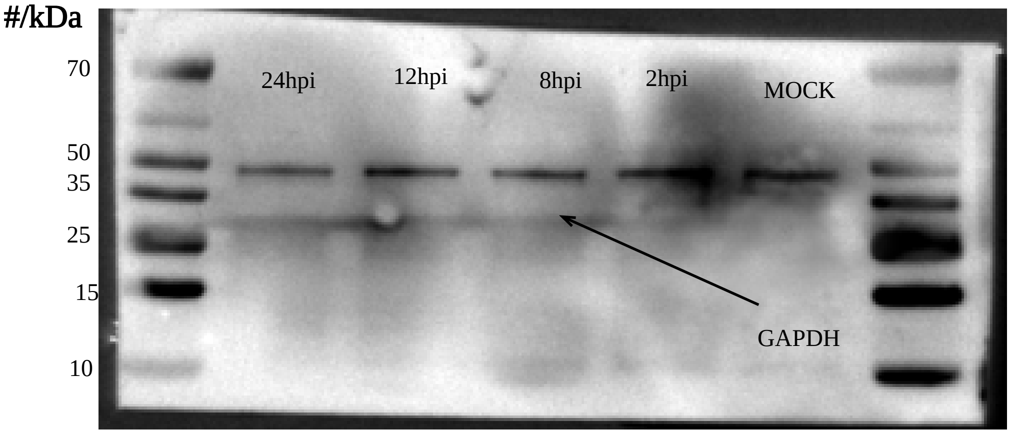
#/kDa
70
24hpi
12hpi
8hpi
2hpi
MOCK
50
35
25
15
GAPDH
10

Fig 1f

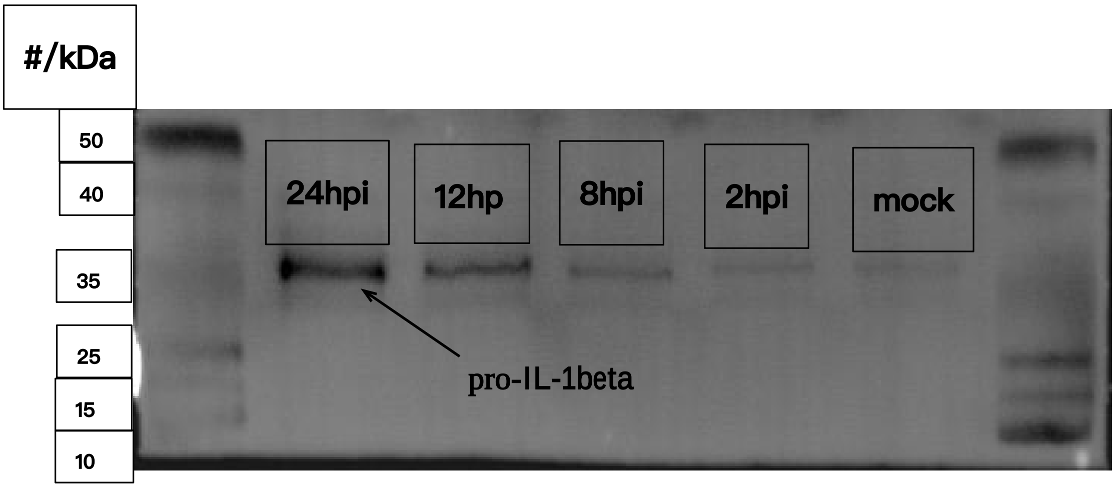
#/kDa
50
40
35
25
15
10
24hpi
12hp
8hpi
2hpi
mock
pro-IL-1beta

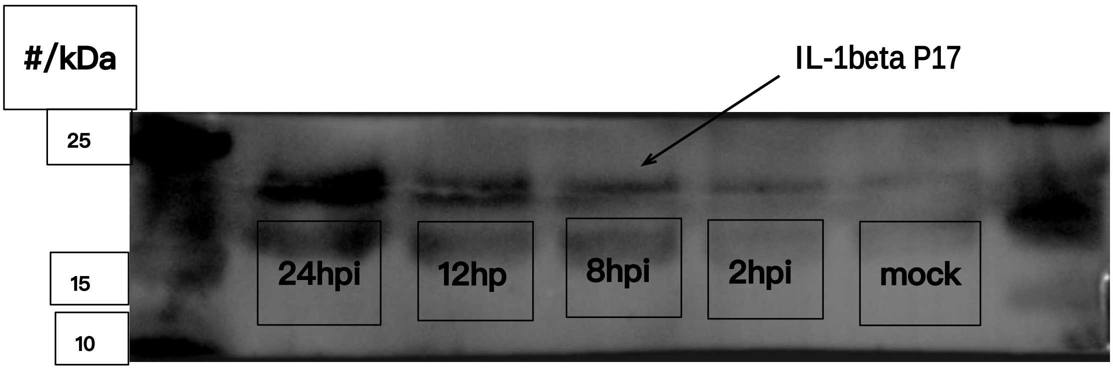
#/kDa
25
15
10
IL-1beta P17
24hpi
12hp
8hpi
2hpi
mock

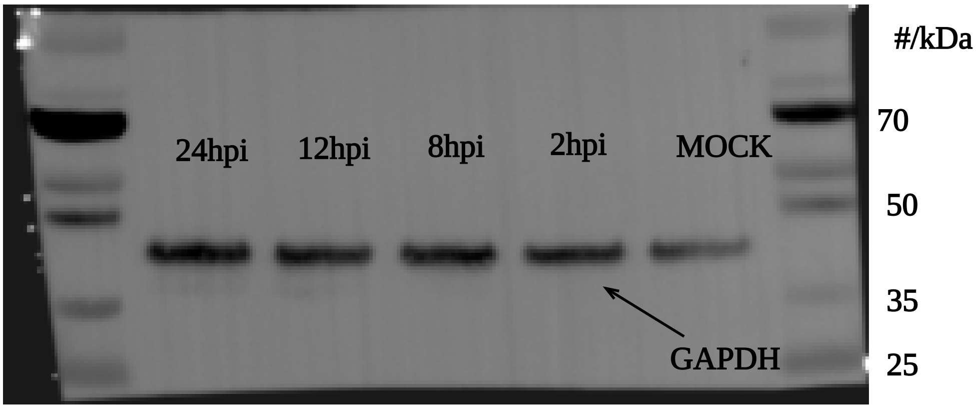
#/kDa
70
50
35
25
24hpi
12hpi
8hpi
2hpi
MOCK
GAPDH

Fig 1 g

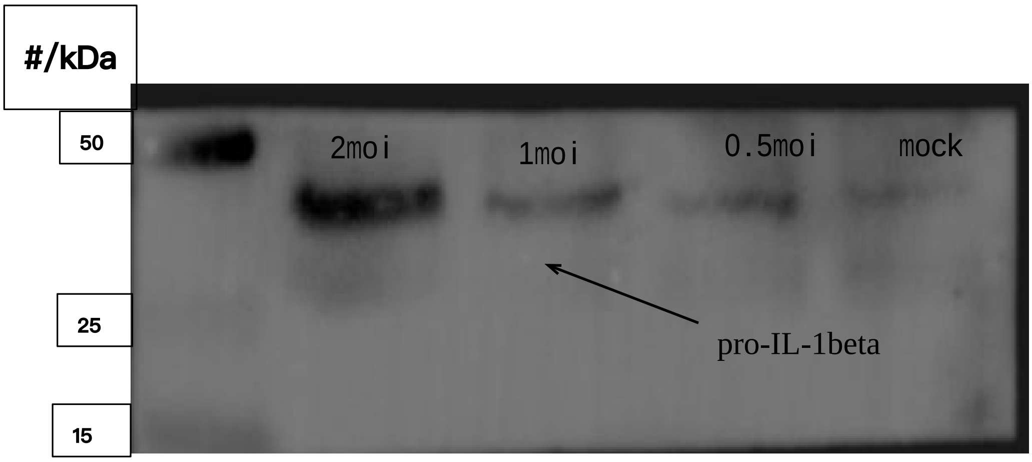
#/kDa
50
25
15
2moi
1moi
0.5moi
mock
pro-IL-1beta

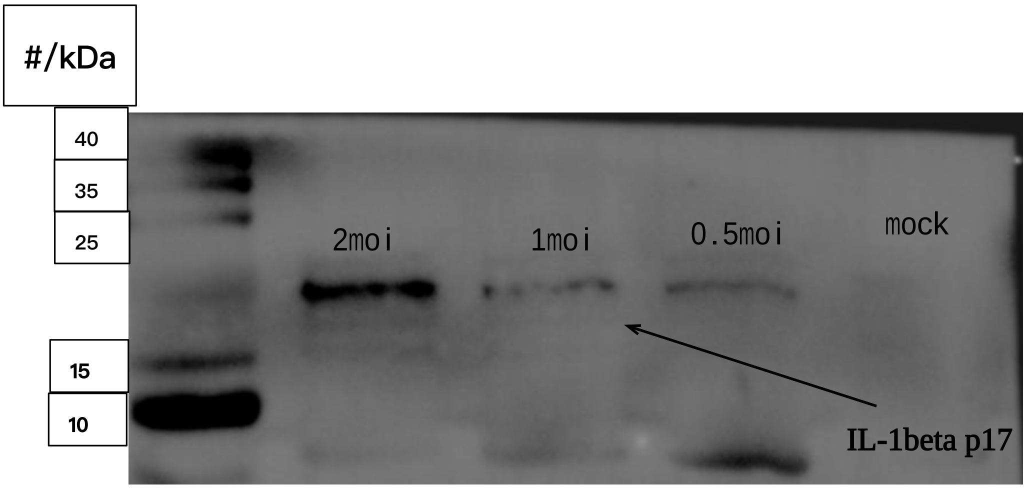
#/kDa
40
35
25
15
10
2moi
1moi
0.5moi
mock
IL-1beta p17

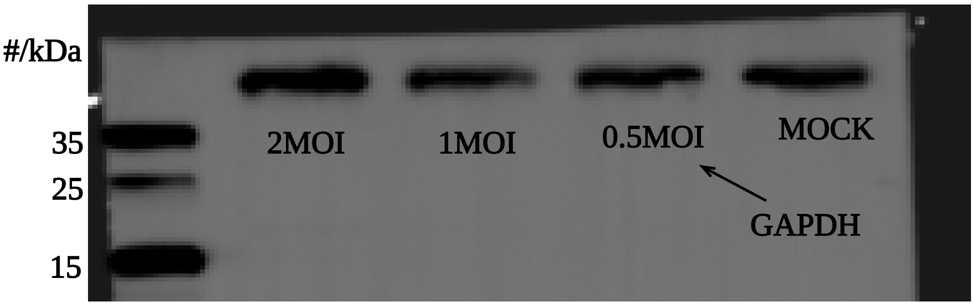
#/kDa
35
25
15
2MOI
1MOI
0.5MOI
MOCK
GAPDH

Fig 2b

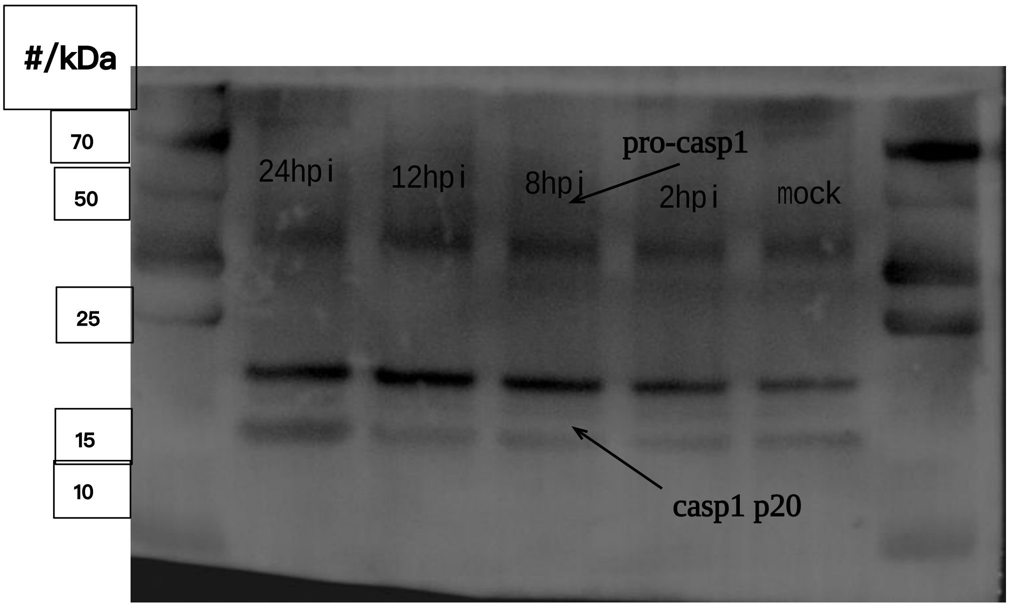
#/kDa
70
50
25
15
10
24hpi
12hpi
8hpi
2hpi
mock
pro-casp1
casp1 p20

Fig 2d

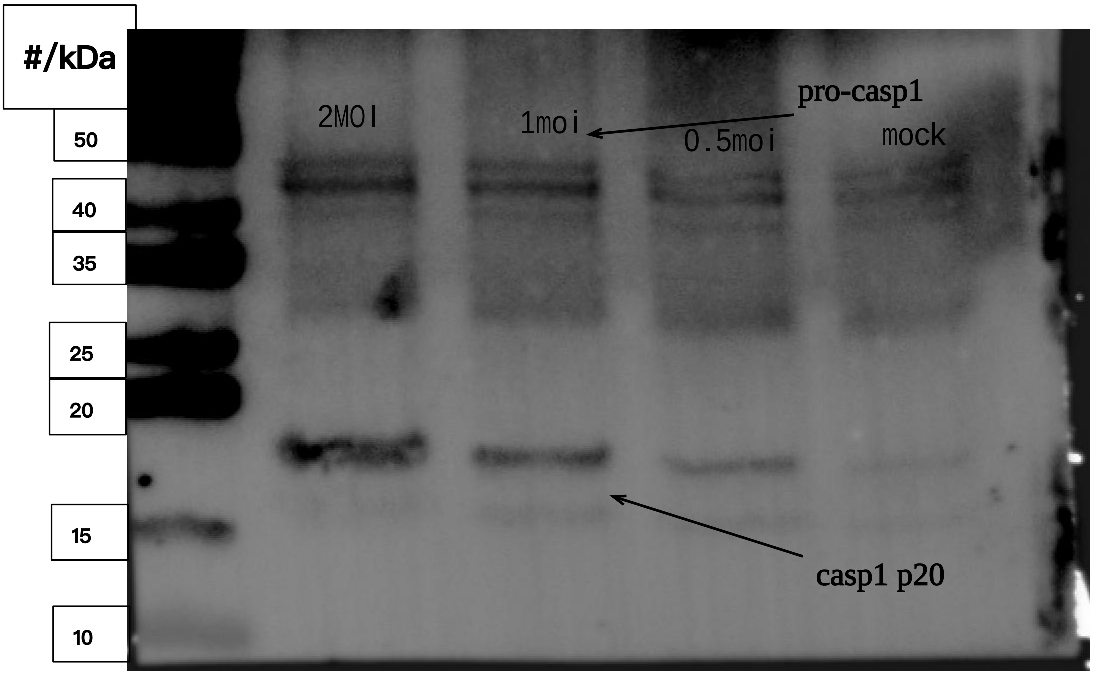
#/kDa
50
40
35
25
20
15
10
2MOI
1moi
0.5moi
mock
pro-casp1
casp1 p20

Fig2b

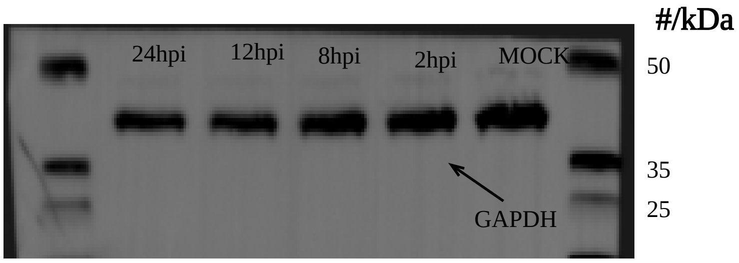
#/kDa
24hpi
12hpi
8hpi
2hpi
MOCK
50
35
25
GAPDH

Fig2d

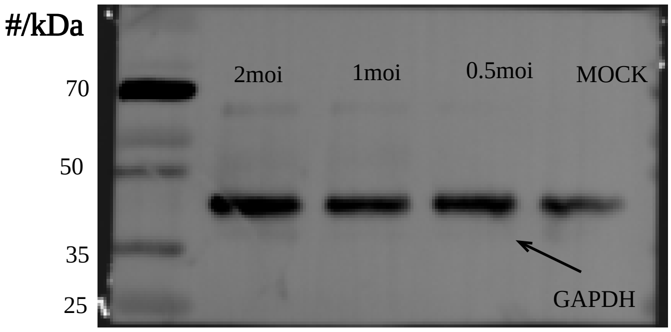
#/kDa
2moi
1moi
0.5moi
MOCK
70
50
35
25
GAPDH

Fig 3 f

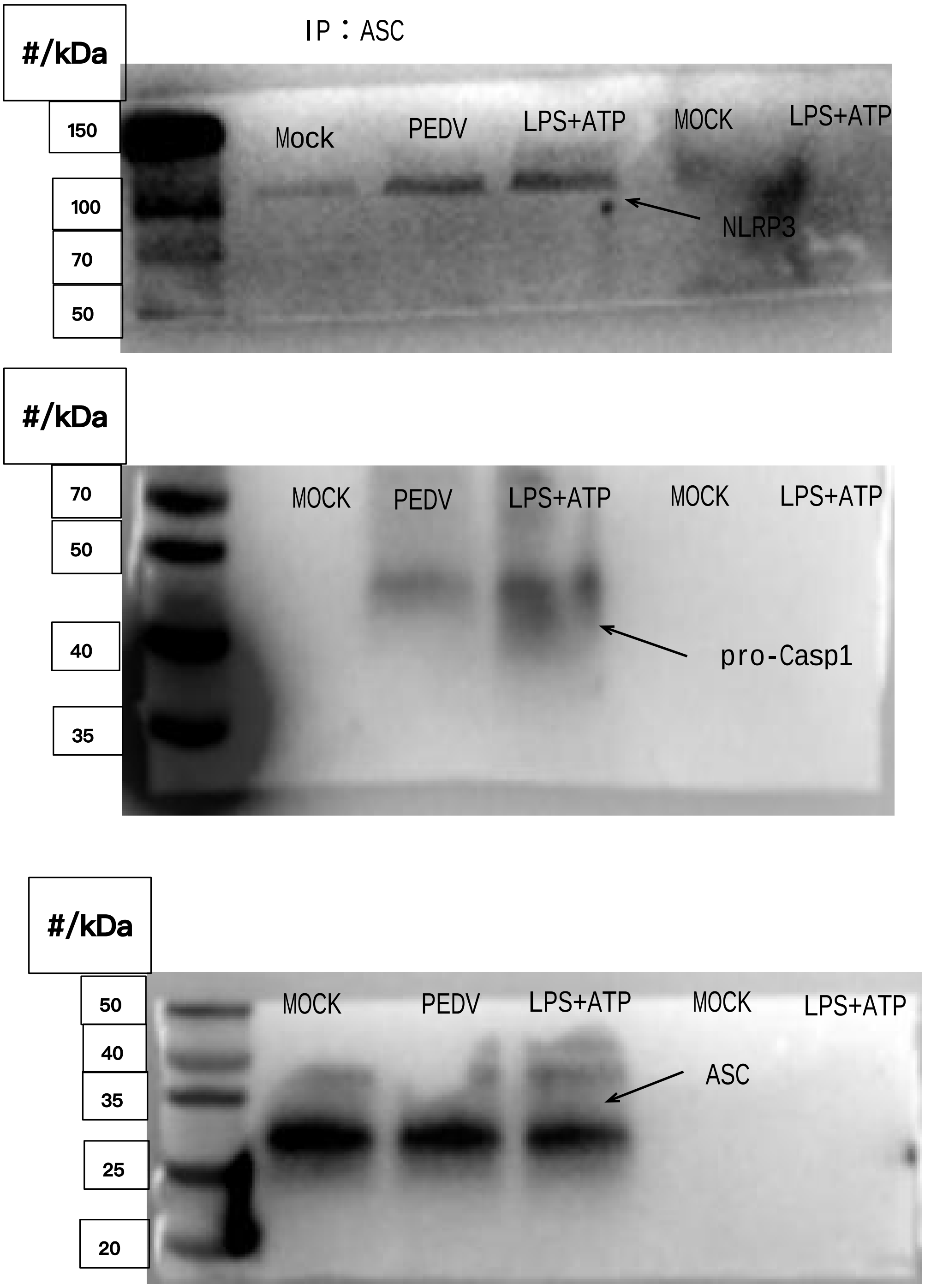
#/kDa
IP：ASC
150
100
70
50
Mock
PEDV
LPS+ATP
MOCK
LPS+ATP
NLRP3
#/kDa
70
50
40
35
MOCK
PEDV
LPS+ATP
MOCK
LPS+ATP
pro-Casp1
#/kDa
50
40
35
25
20
MOCK
PEDV
LPS+ATP
MOCK
LPS+ATP
ASC

Fig 3 e

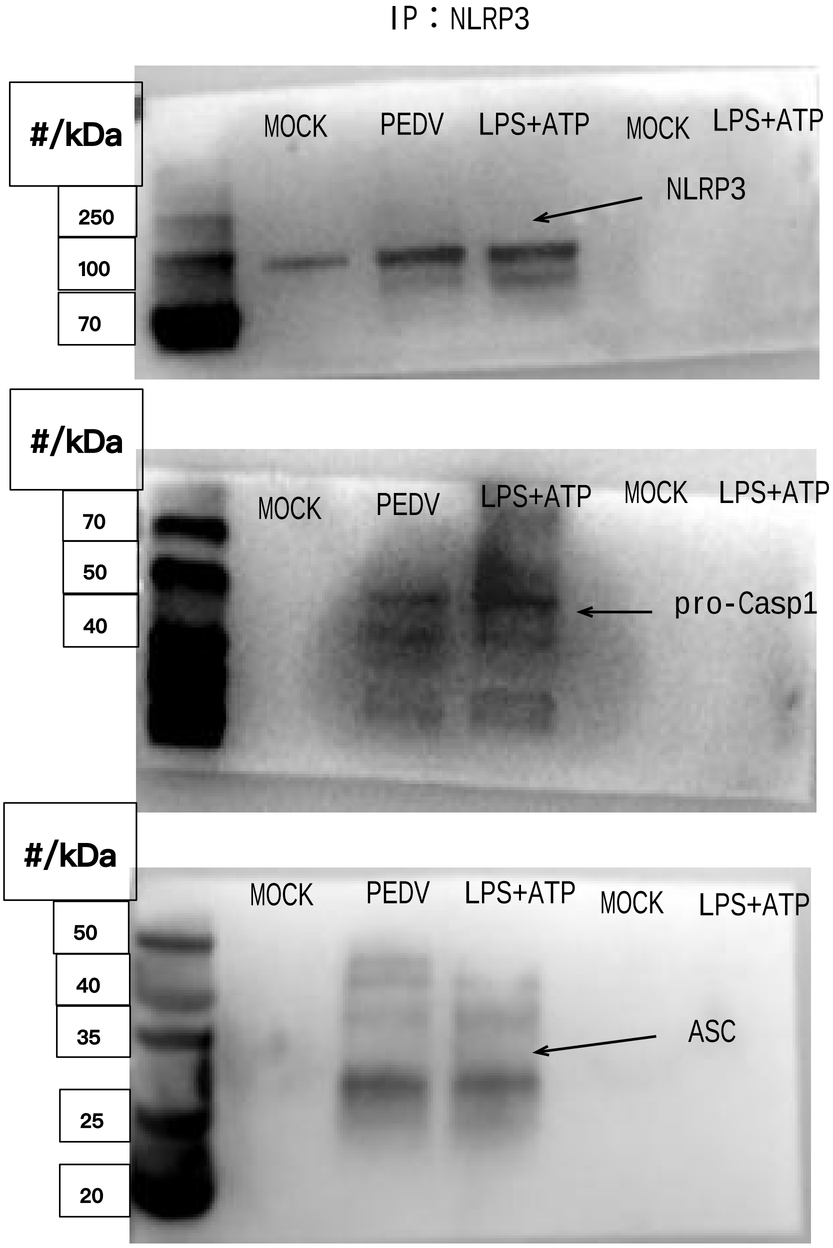
IP：NLRP3
#/kDa
250
100
70
MOCK
PEDV
LPS+ATP
MOCK
LPS+ATP
NLRP3
#/kDa
70
50
40
MOCK
PEDV
LPS+ATP
MOCK
LPS+ATP
pro-Casp1
#/kDa
50
40
35
25
20
MOCK
PEDV
LPS+ATP
MOCK
LPS+ATP
ASC

Fig 4e

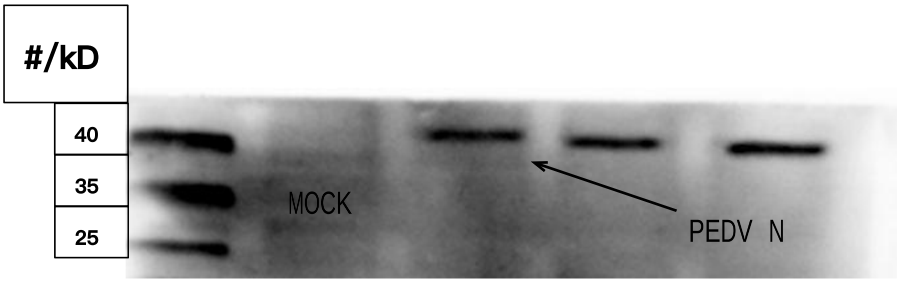
#/kD
40
35
25
MOCK
PEDV N

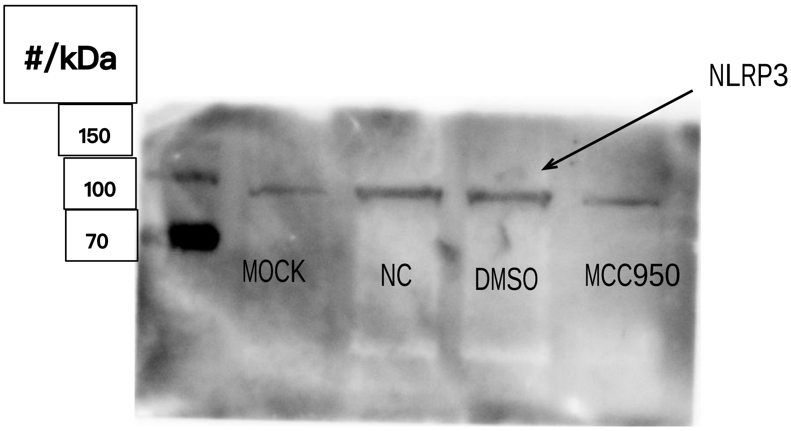
#/kDa
NLRP3
150
100
70
MOCK
NC
DMSO
MCC950

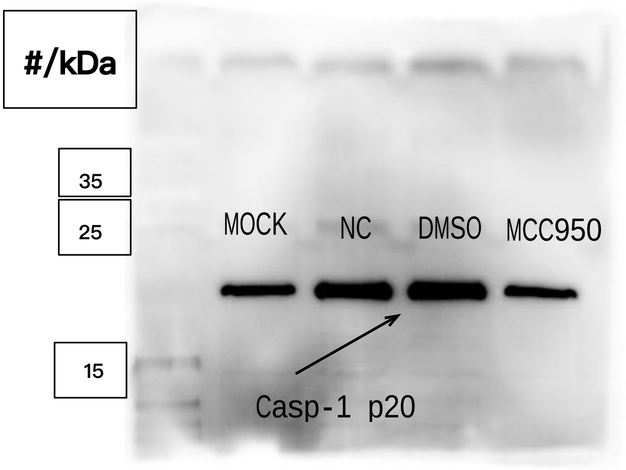
#/kDa
35
25
MOCK
NC
DMSO
MCC950
15
Casp-1 p20

Fig 4e

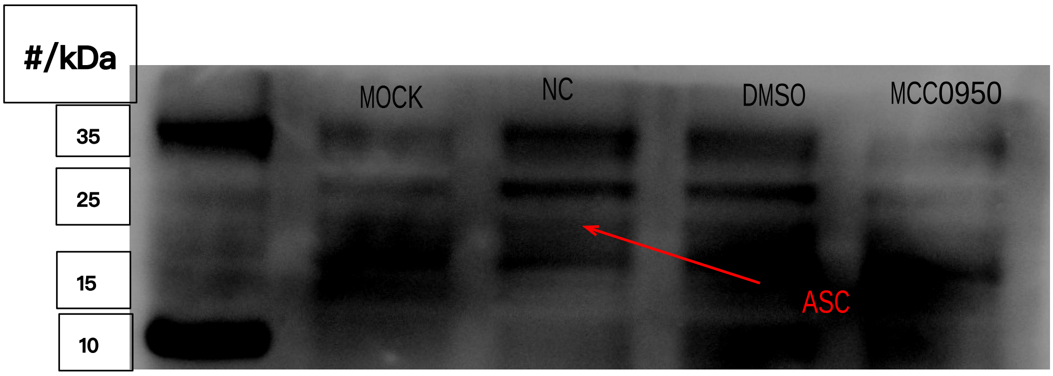
#/kDa
35
25
15
10
MOCK
NC
DMSO
MCC0950
ASC

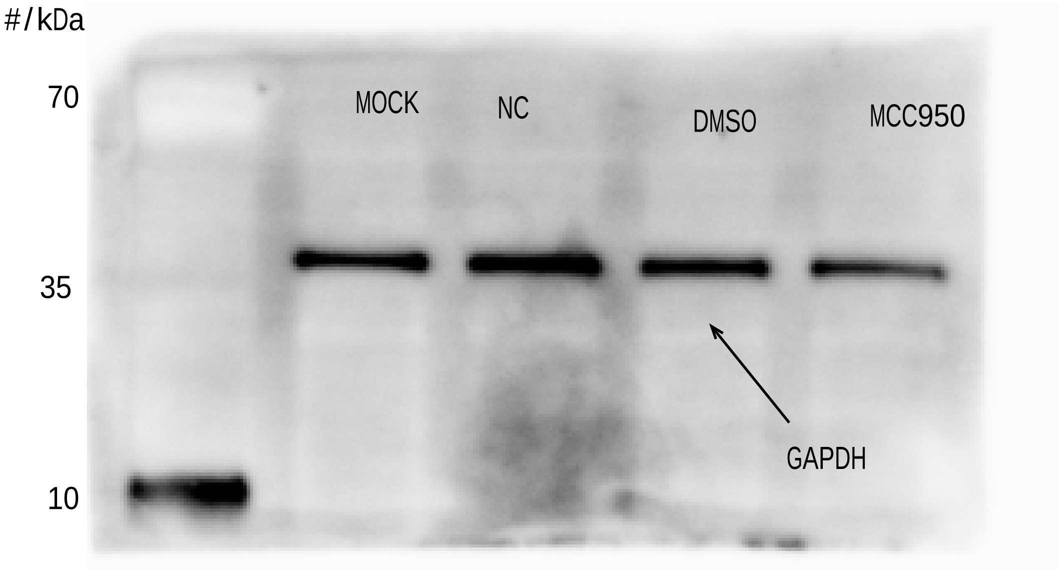
#/kDa
70
35
10
MOCK
NC
DMSO
MCC950
GAPDH

Fig 4f

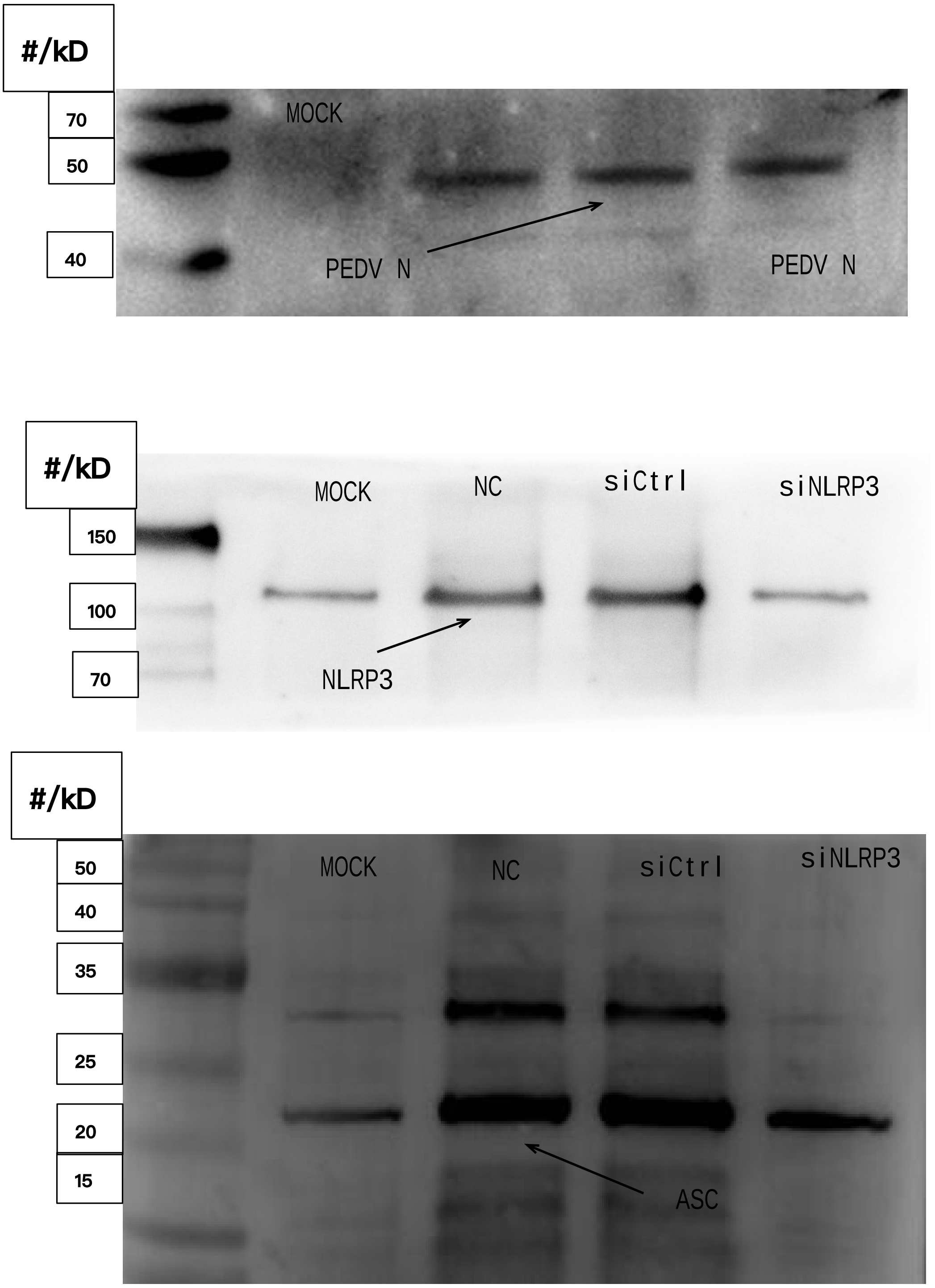
#/kD
70
50
40
MOCK
PEDV N
PEDV N
#/kD
150
100
70
MOCK
NC
siCtrl
siNLRP3
NLRP3
#/kD
50
40
35
25
20
15
MOCK
NC
siCtrl
siNLRP3
ASC

Fig 4f

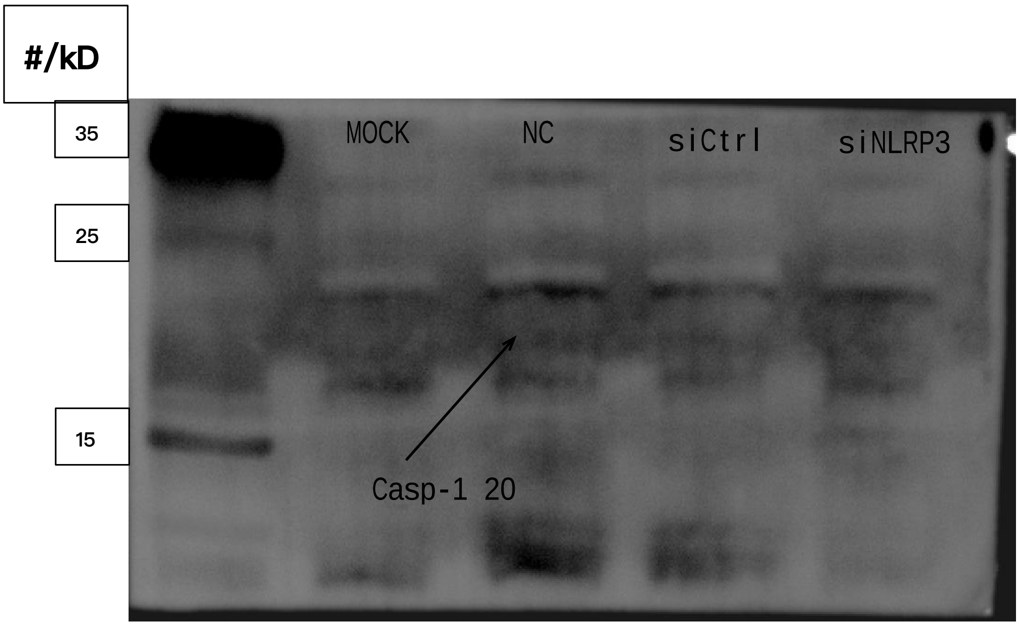
#/kD
35
25
15
MOCK
NC
siCtrl
siNLRP3
Casp-1 20

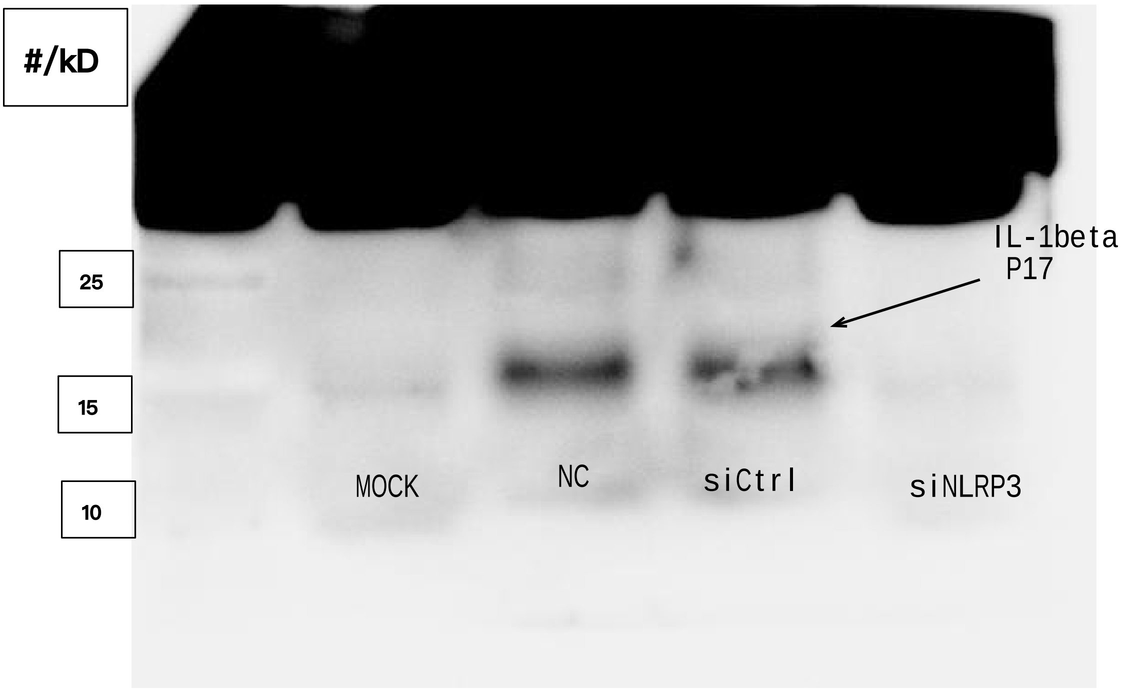
#/kD
25
15
10
IL-1beta
P17
MOCK
NC
siCtrl
siNLRP3

Fig 4f

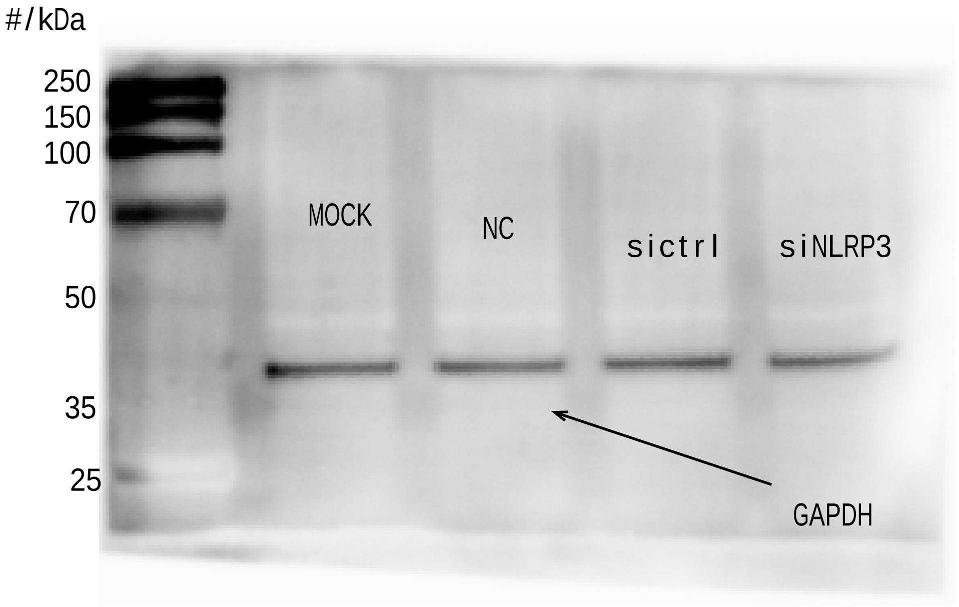

Fig 6a

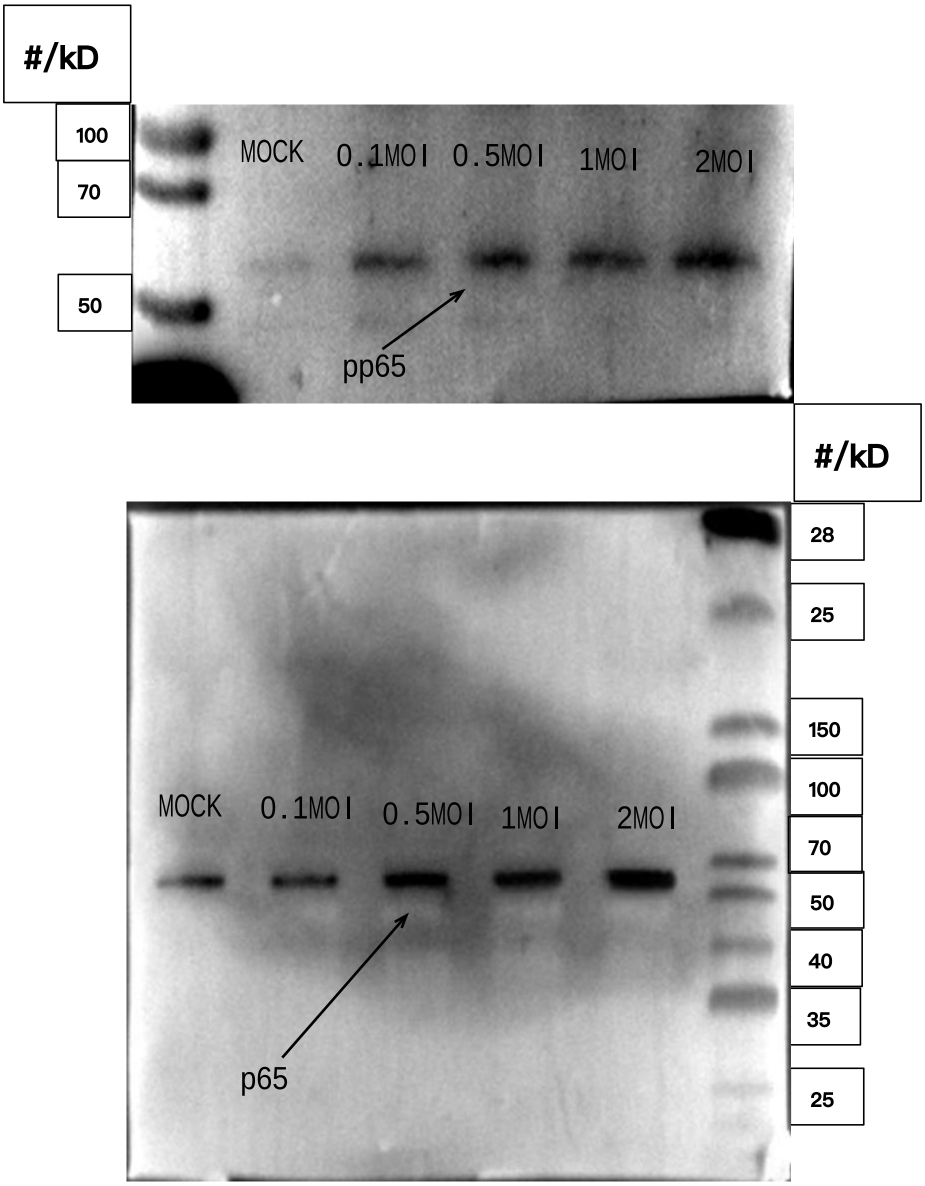
#/kD
100
70
50
MOCK
0.1MOI
0.5MOI
1MOI
2MOI
pp65
#/kD
28
25
150
100
70
50
40
35
25
MOCK
0.1MOI
0.5MOI
1MOI
2MOI
p65

Fig6a

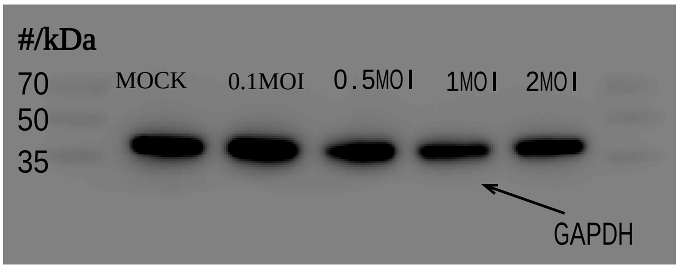

Fig 6d

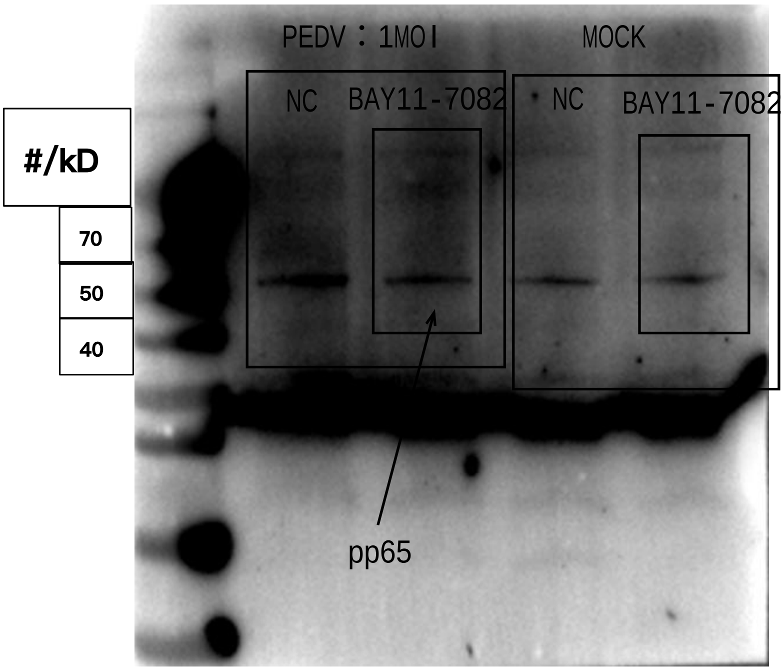

PEDV：1MOI
MOCK
NC
BAY11-7082
NC
BAY11-7082
#/kD
70
50
40
pp65

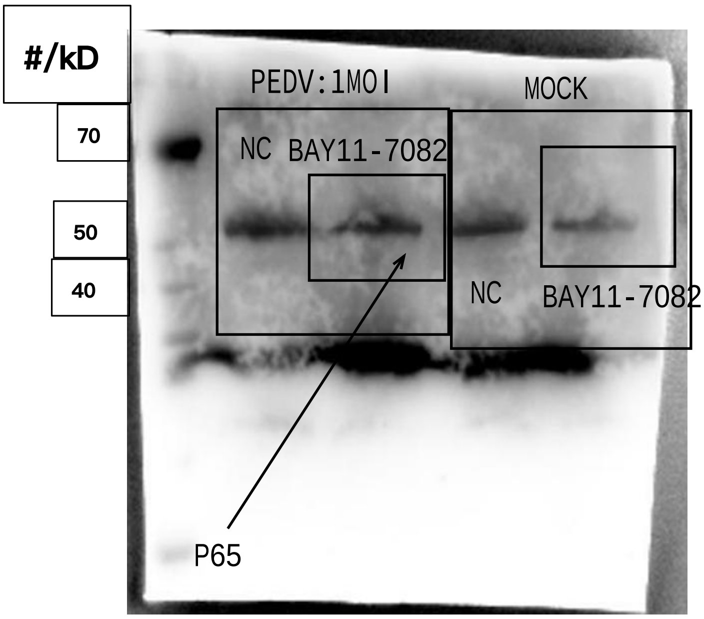

#/kD
70
50
40
PEDV:1MOI
MOCK
NC
BAY11-7082
NC
BAY11-7082
P65

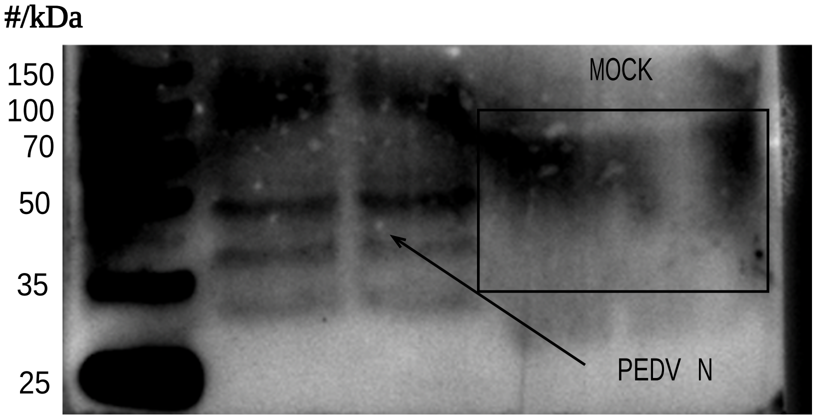
#/kDa
150
100
70
50
35
25
MOCK
PEDV N

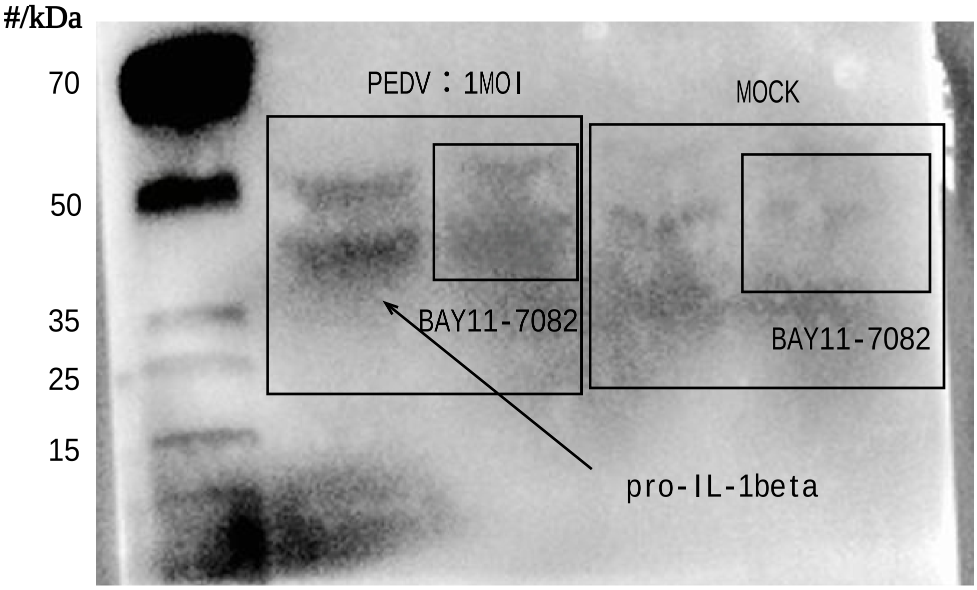
#/kDa
70
50
35
25
15
PEDV：1MOI
MOCK
BAY11-7082
BAY11-7082
pro-IL-1beta

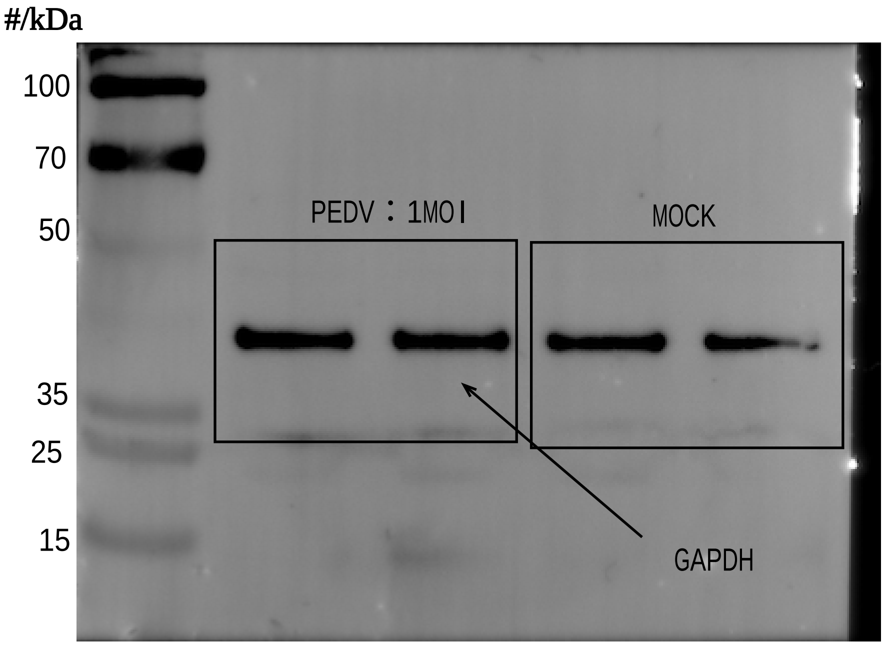
#/kDa
100
70
50
35
25
15
PEDV：1MOI
MOCK
GAPDH

Fig 7e

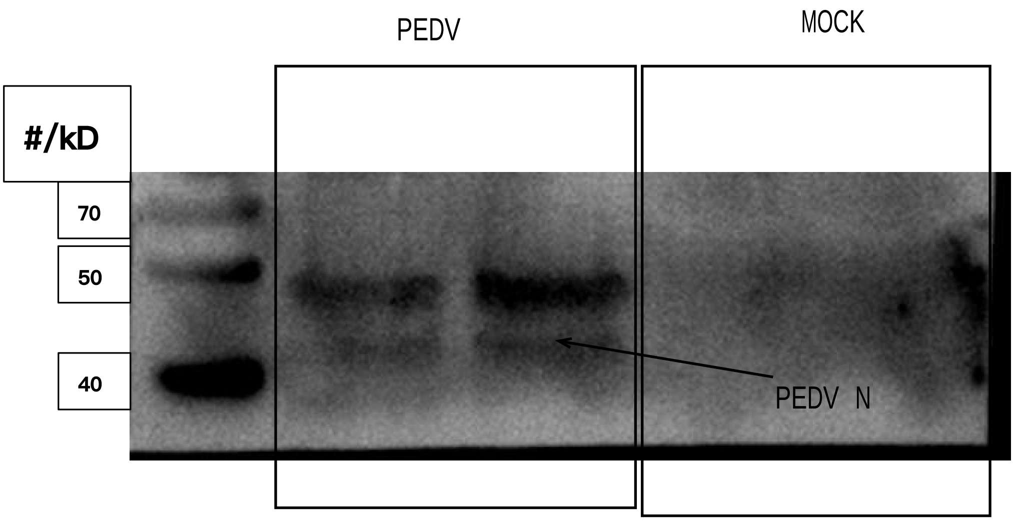
PEDV
MOCK
#/kD
70
50
40
PEDV N

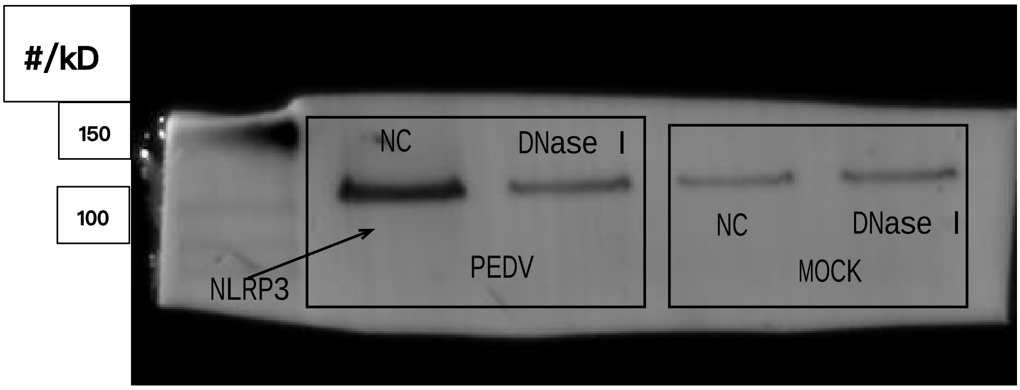
#/kD
150
100
NC
DNase I
PEDV
NLRP3
NC
DNase I
MOCK

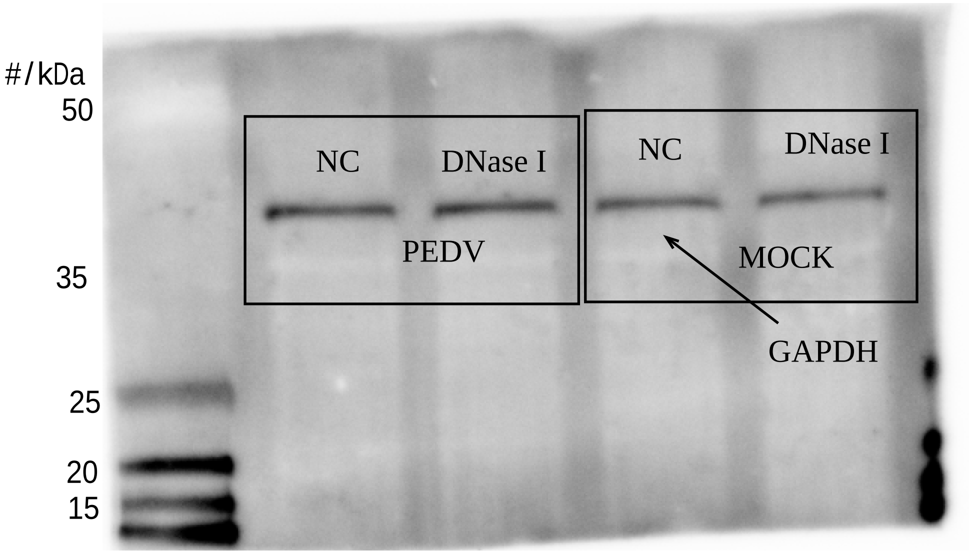
#/kDa
50
35
25
20
15
NC
DNase I
PEDV
NC
DNase I
MOCK
GAPDH
