# Supplementary figures and images for: Porcine Epidemic Diarrhea Virus Infection of Porcine Intestinal Epithelial Cells Causes Mitochondrial DNA Release and the Activation of the NLRP3 Inflammasome to Mediate Interleukin-1β Secretion
Source: Vet Sci. 2024 Dec 12;11(12):643. doi: 10.3390/vetsci11120643 (PMC11680147; doi:10.3390/vetsci11120643)

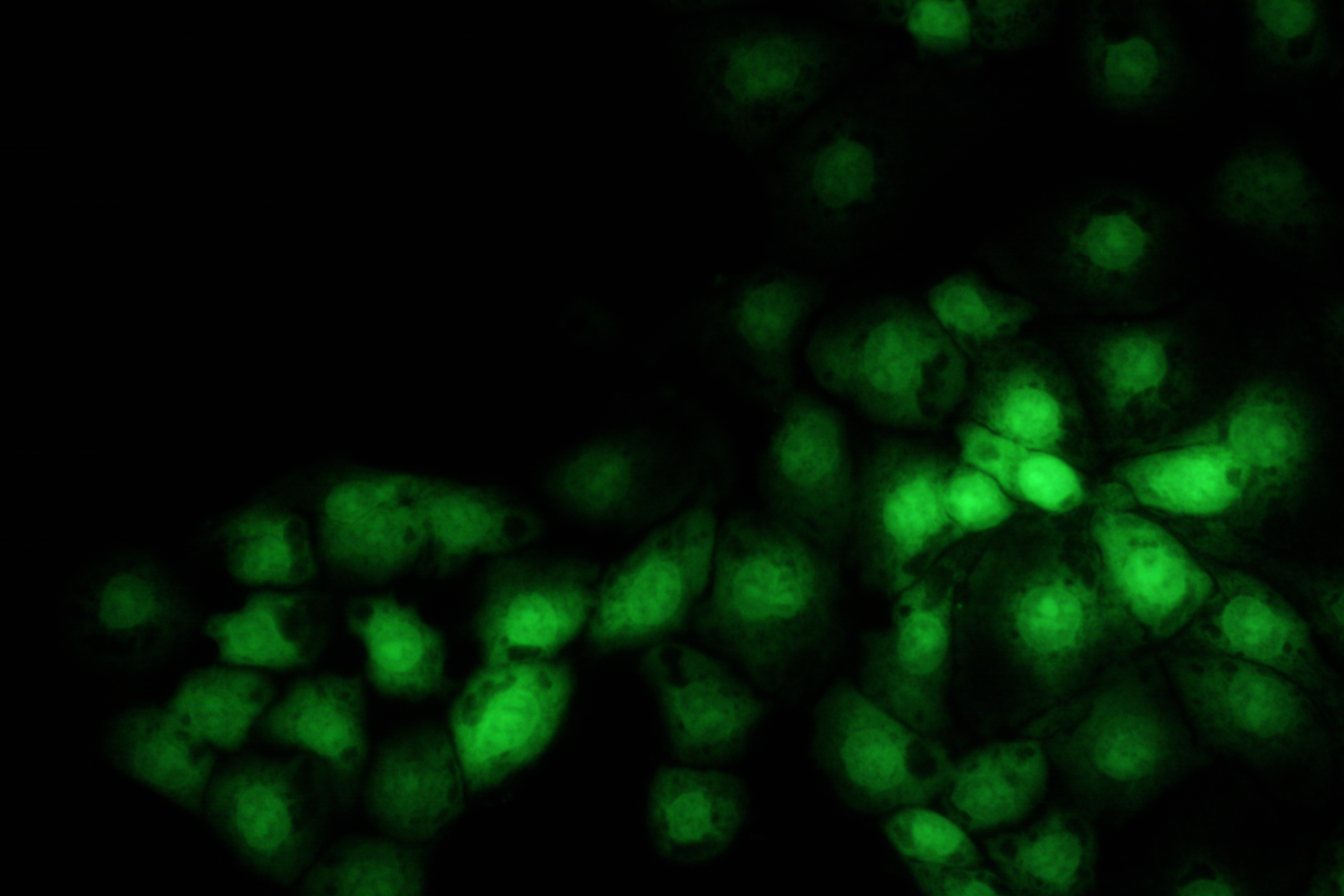

Supplement: Supplementary file 1 [file vetsci-11-00643-s001.zip › Supplementary Materials/0001(1).tif]

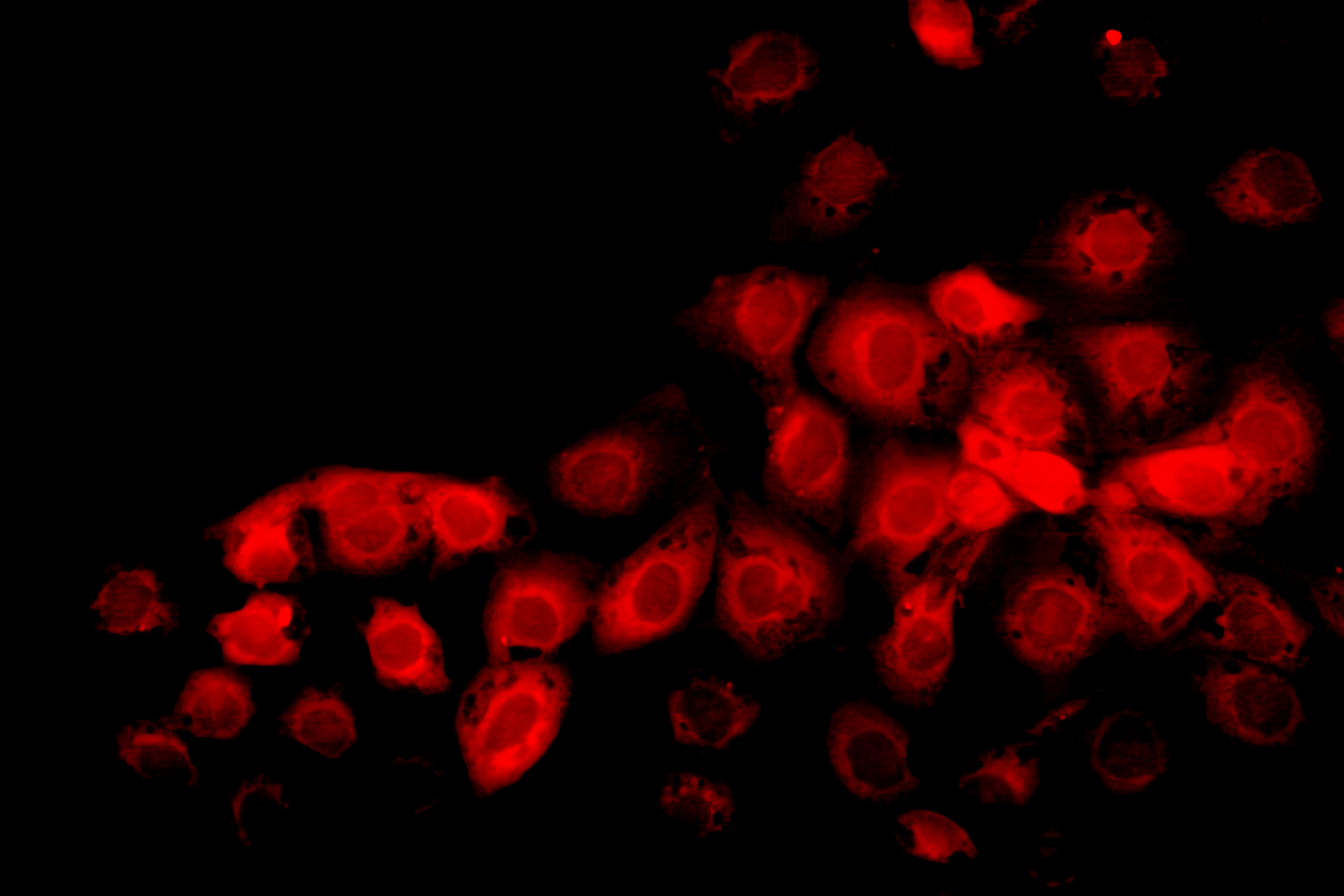

Supplement: Supplementary file 1 [file vetsci-11-00643-s001.zip › Supplementary Materials/0002(1).tif]

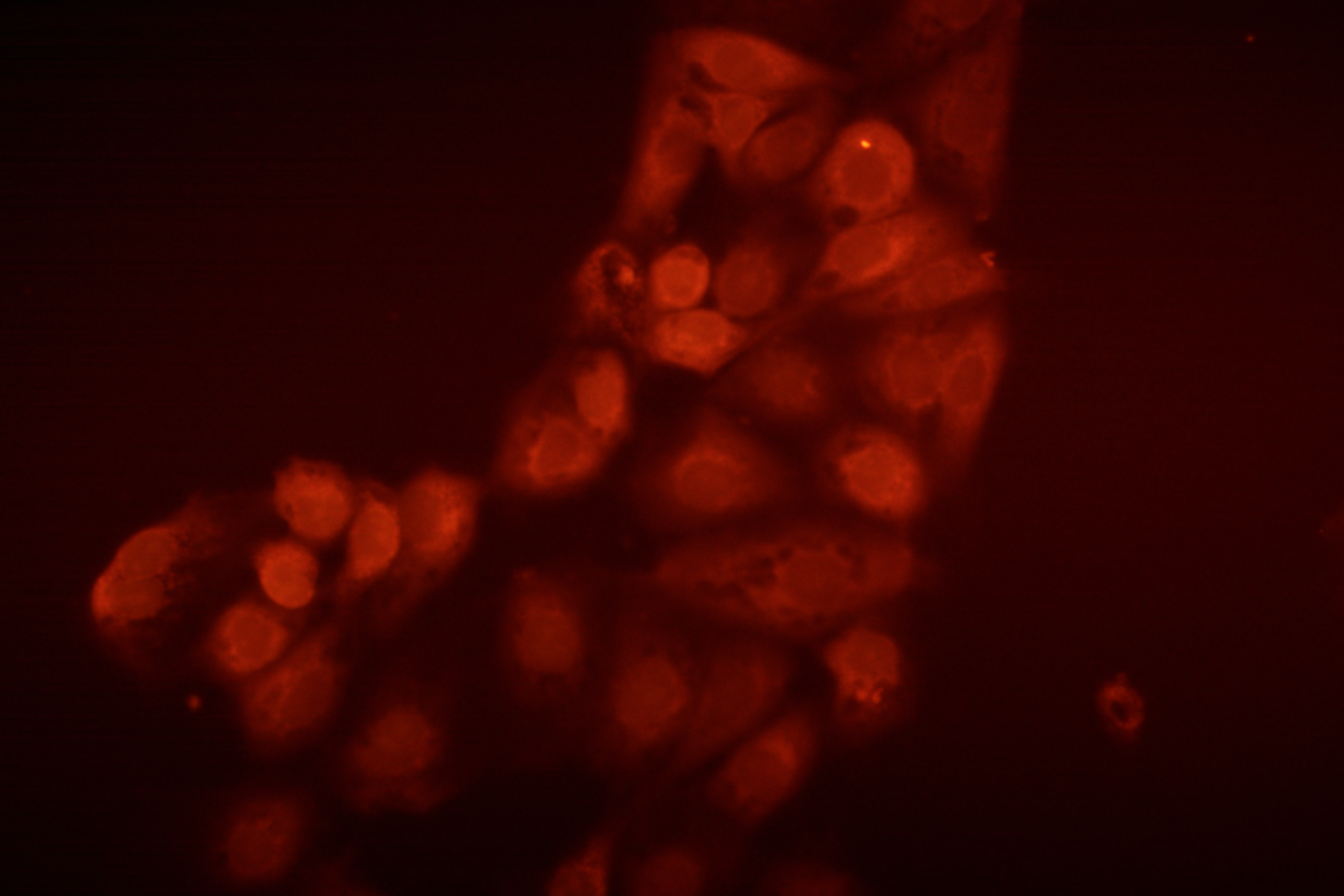

Supplement: Supplementary file 1 [file vetsci-11-00643-s001.zip › Supplementary Materials/0012 (2)(1).tif]

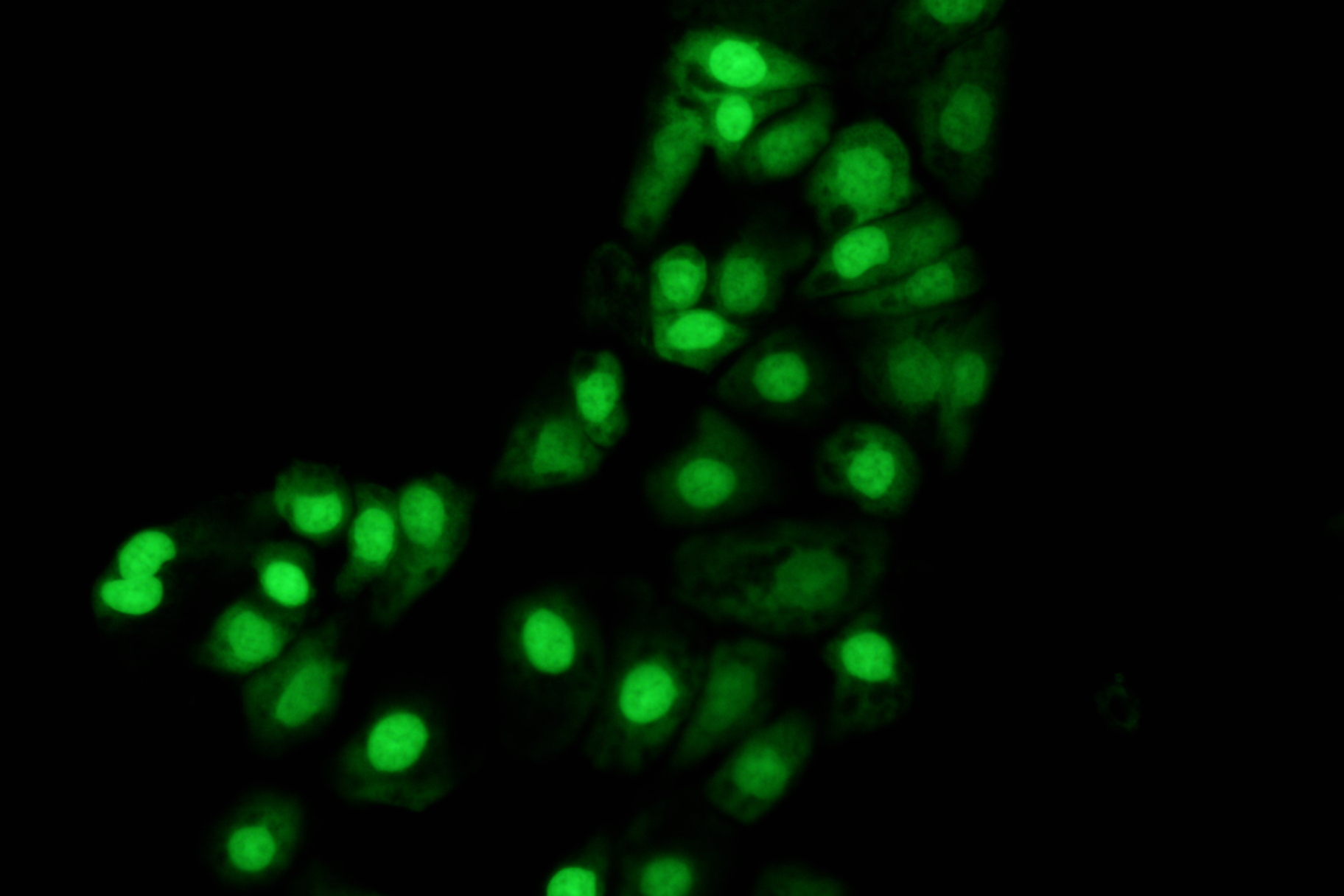

Supplement: Supplementary file 1 [file vetsci-11-00643-s001.zip › Supplementary Materials/0013 (2)(1).tif]

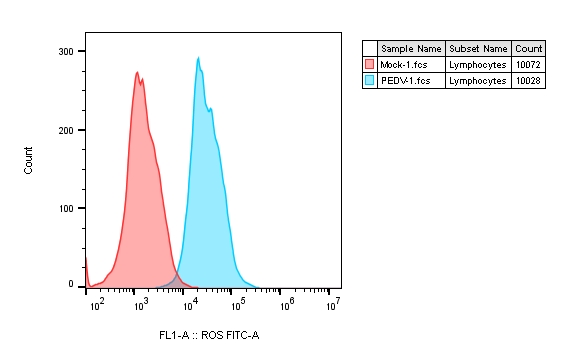

Supplement: Supplementary file 1 [file vetsci-11-00643-s001.zip › Supplementary Materials/Original record of fluorescence intensity analysis of flow cytometry (n=3)/n=3/Composite image.jpg]

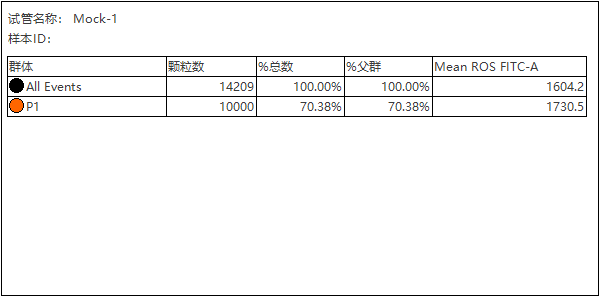

Supplement: Supplementary file 1 [file vetsci-11-00643-s001.zip › Supplementary Materials/Original record of fluorescence intensity analysis of flow cytometry (n=3)/n=3/Fluorescence intensity/Mock-1_Statistics1.bmp]

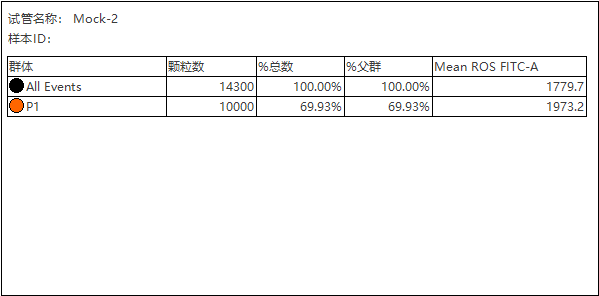

Supplement: Supplementary file 1 [file vetsci-11-00643-s001.zip › Supplementary Materials/Original record of fluorescence intensity analysis of flow cytometry (n=3)/n=3/Fluorescence intensity/Mock-2_Statistics1.bmp]

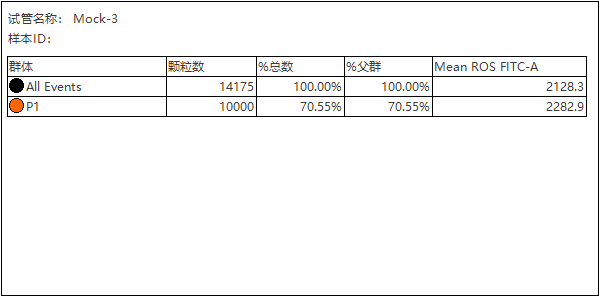

Supplement: Supplementary file 1 [file vetsci-11-00643-s001.zip › Supplementary Materials/Original record of fluorescence intensity analysis of flow cytometry (n=3)/n=3/Fluorescence intensity/Mock-3_Statistics1.bmp]

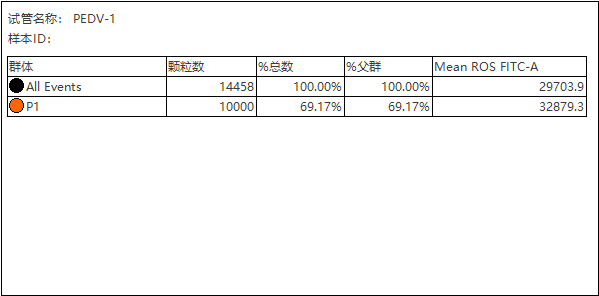

Supplement: Supplementary file 1 [file vetsci-11-00643-s001.zip › Supplementary Materials/Original record of fluorescence intensity analysis of flow cytometry (n=3)/n=3/Fluorescence intensity/PEDV-1_Statistics1.bmp]

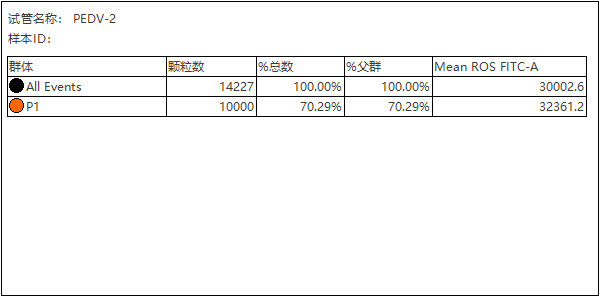

Supplement: Supplementary file 1 [file vetsci-11-00643-s001.zip › Supplementary Materials/Original record of fluorescence intensity analysis of flow cytometry (n=3)/n=3/Fluorescence intensity/PEDV-2_Statistics1.bmp]

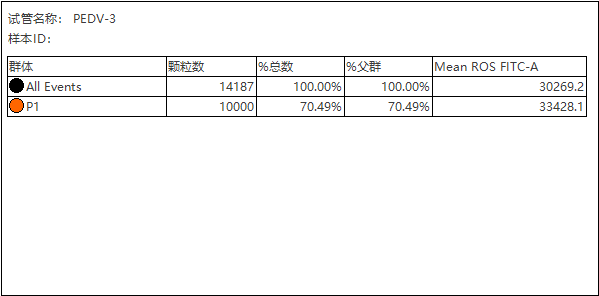

Supplement: Supplementary file 1 [file vetsci-11-00643-s001.zip › Supplementary Materials/Original record of fluorescence intensity analysis of flow cytometry (n=3)/n=3/Fluorescence intensity/PEDV-3_Statistics1.bmp]

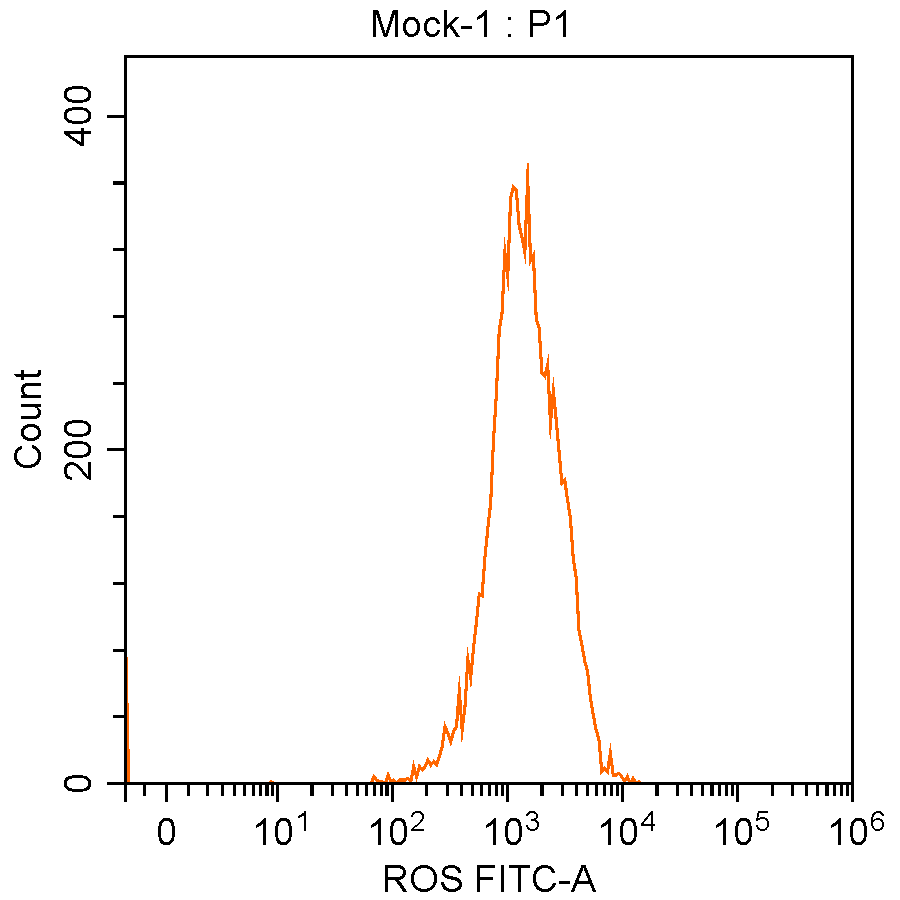

Supplement: Supplementary file 1 [file vetsci-11-00643-s001.zip › Supplementary Materials/Original record of fluorescence intensity analysis of flow cytometry (n=3)/n=3/Mock-1_Plot1.bmp]

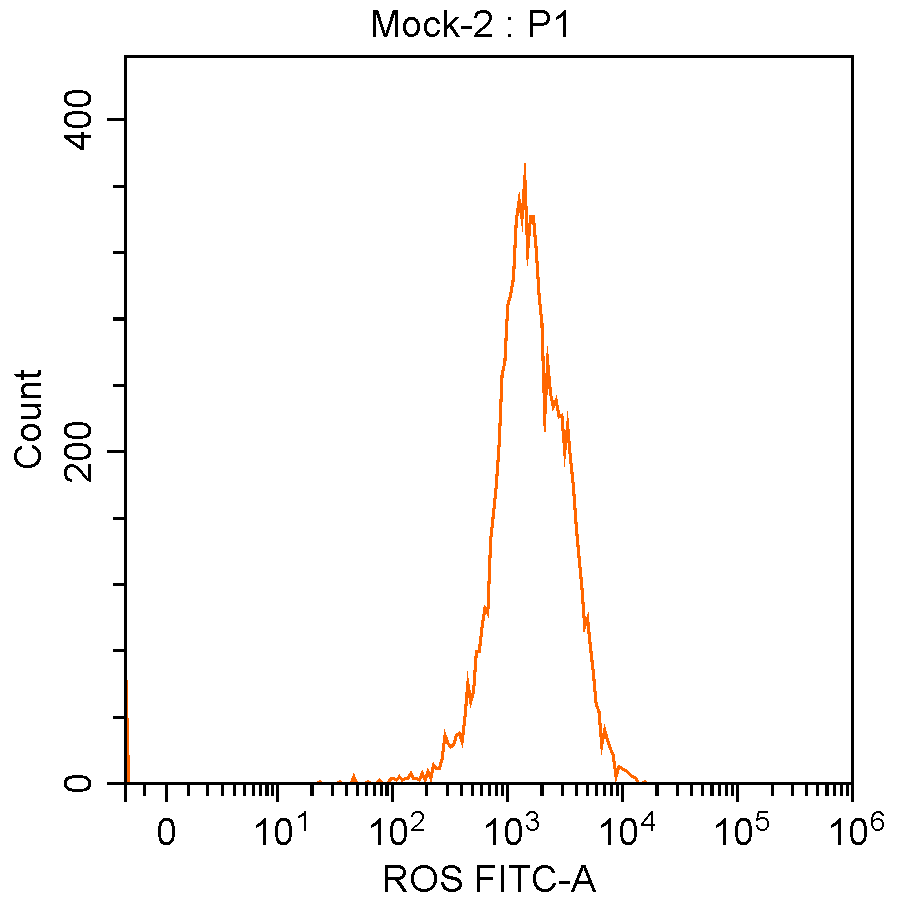

Supplement: Supplementary file 1 [file vetsci-11-00643-s001.zip › Supplementary Materials/Original record of fluorescence intensity analysis of flow cytometry (n=3)/n=3/Mock-2_Plot1.bmp]

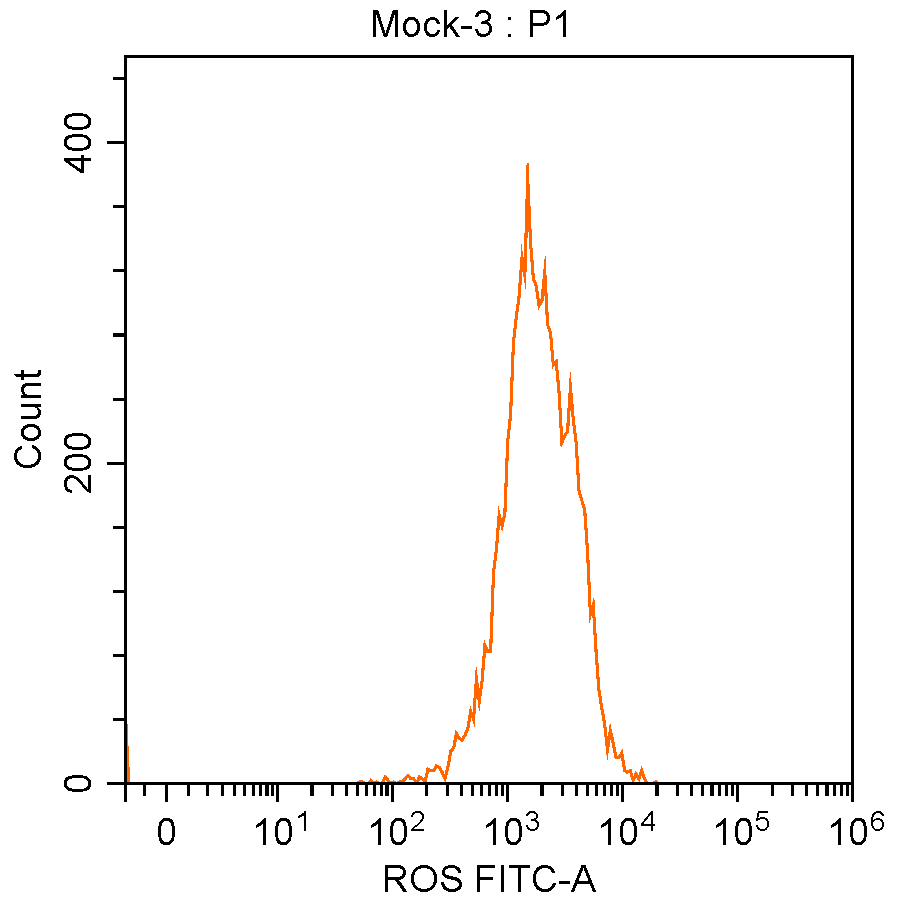

Supplement: Supplementary file 1 [file vetsci-11-00643-s001.zip › Supplementary Materials/Original record of fluorescence intensity analysis of flow cytometry (n=3)/n=3/Mock-3_Plot1.bmp]

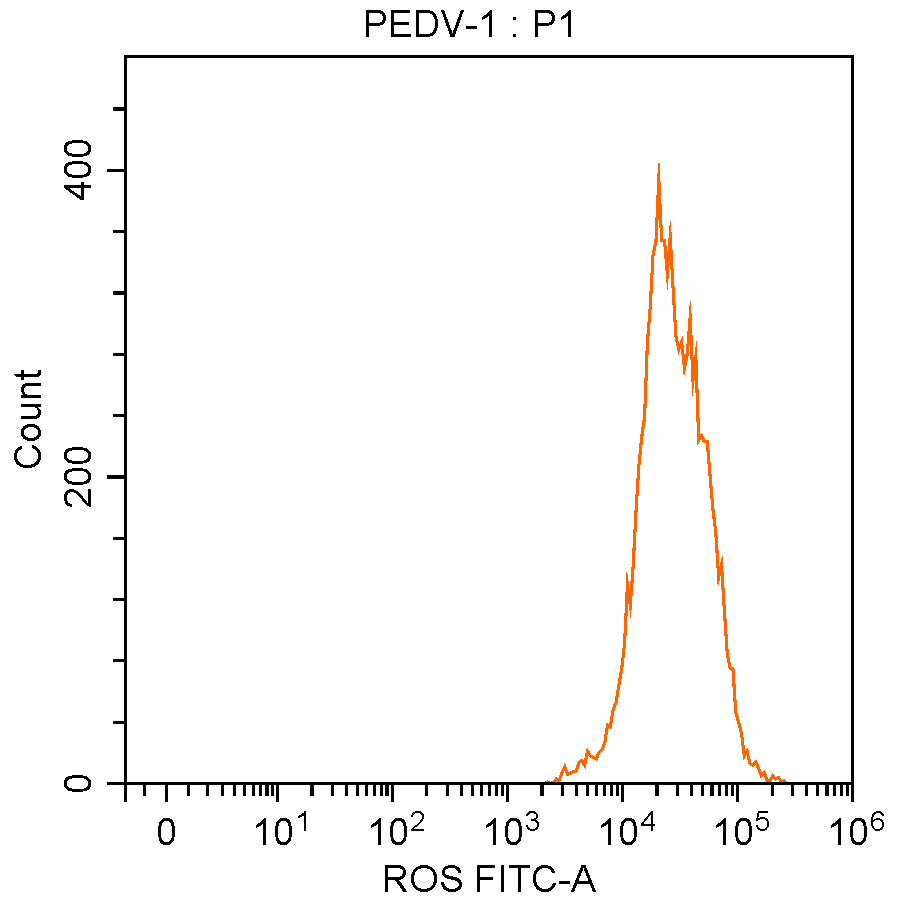

Supplement: Supplementary file 1 [file vetsci-11-00643-s001.zip › Supplementary Materials/Original record of fluorescence intensity analysis of flow cytometry (n=3)/n=3/PEDV-1_Plot1.bmp]

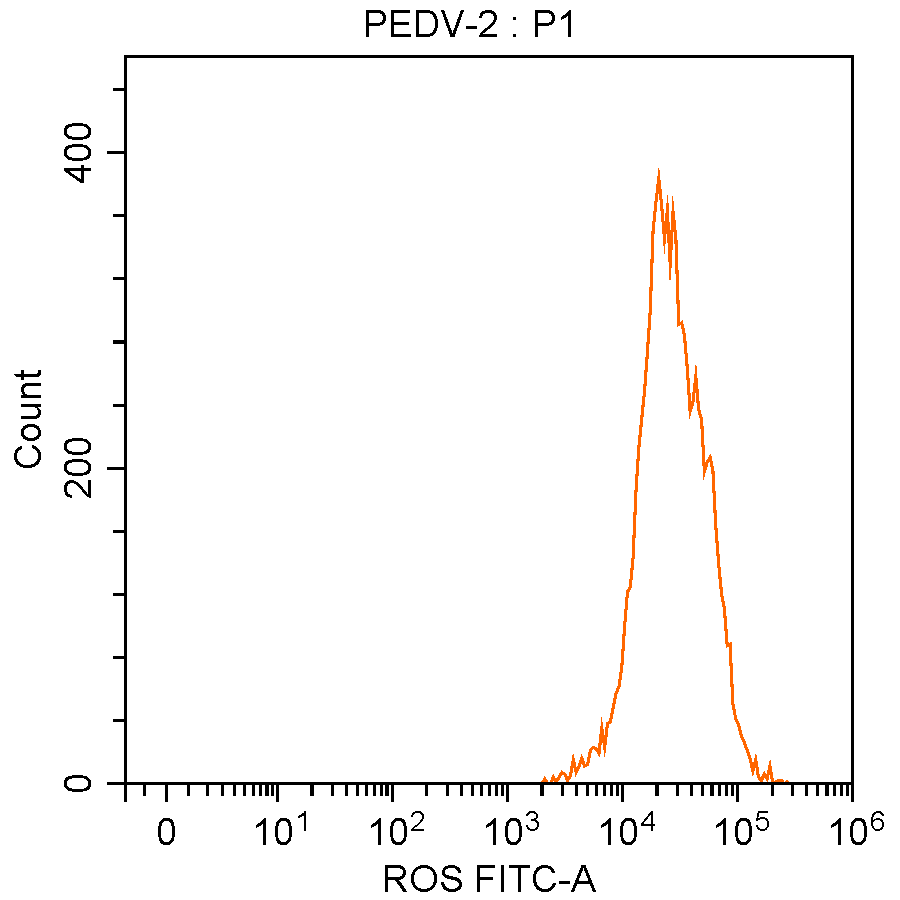

Supplement: Supplementary file 1 [file vetsci-11-00643-s001.zip › Supplementary Materials/Original record of fluorescence intensity analysis of flow cytometry (n=3)/n=3/PEDV-2_Plot1.bmp]

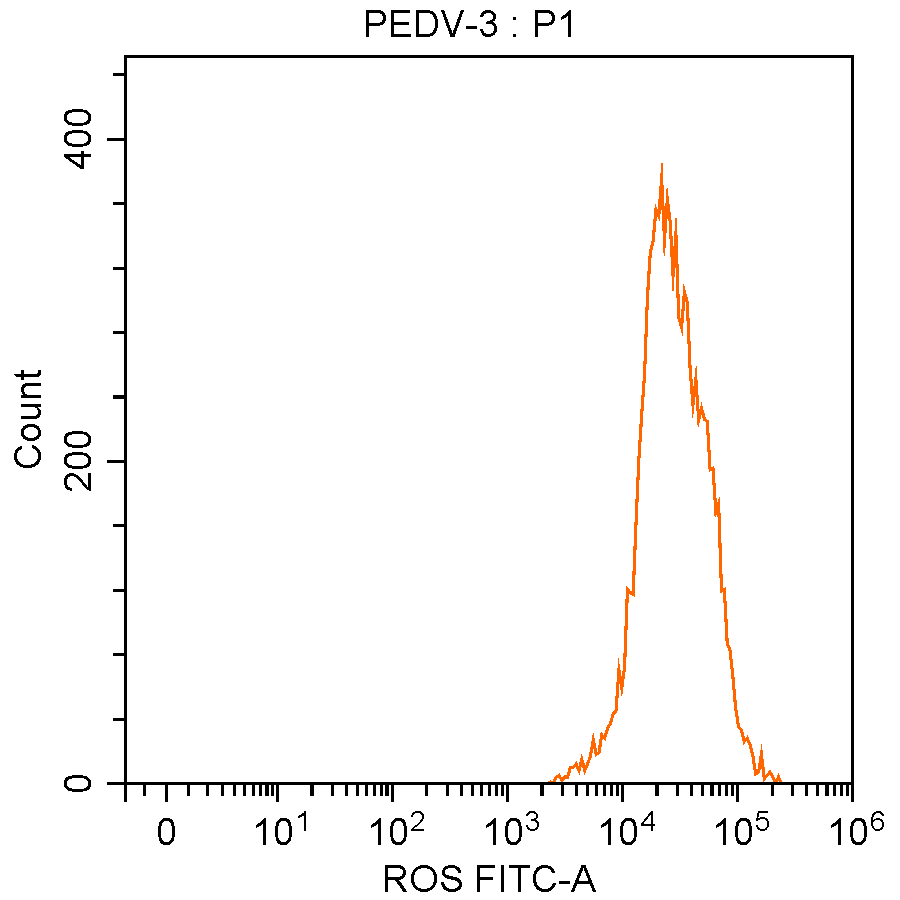

Supplement: Supplementary file 1 [file vetsci-11-00643-s001.zip › Supplementary Materials/Original record of fluorescence intensity analysis of flow cytometry (n=3)/n=3/PEDV-3_Plot1.bmp]

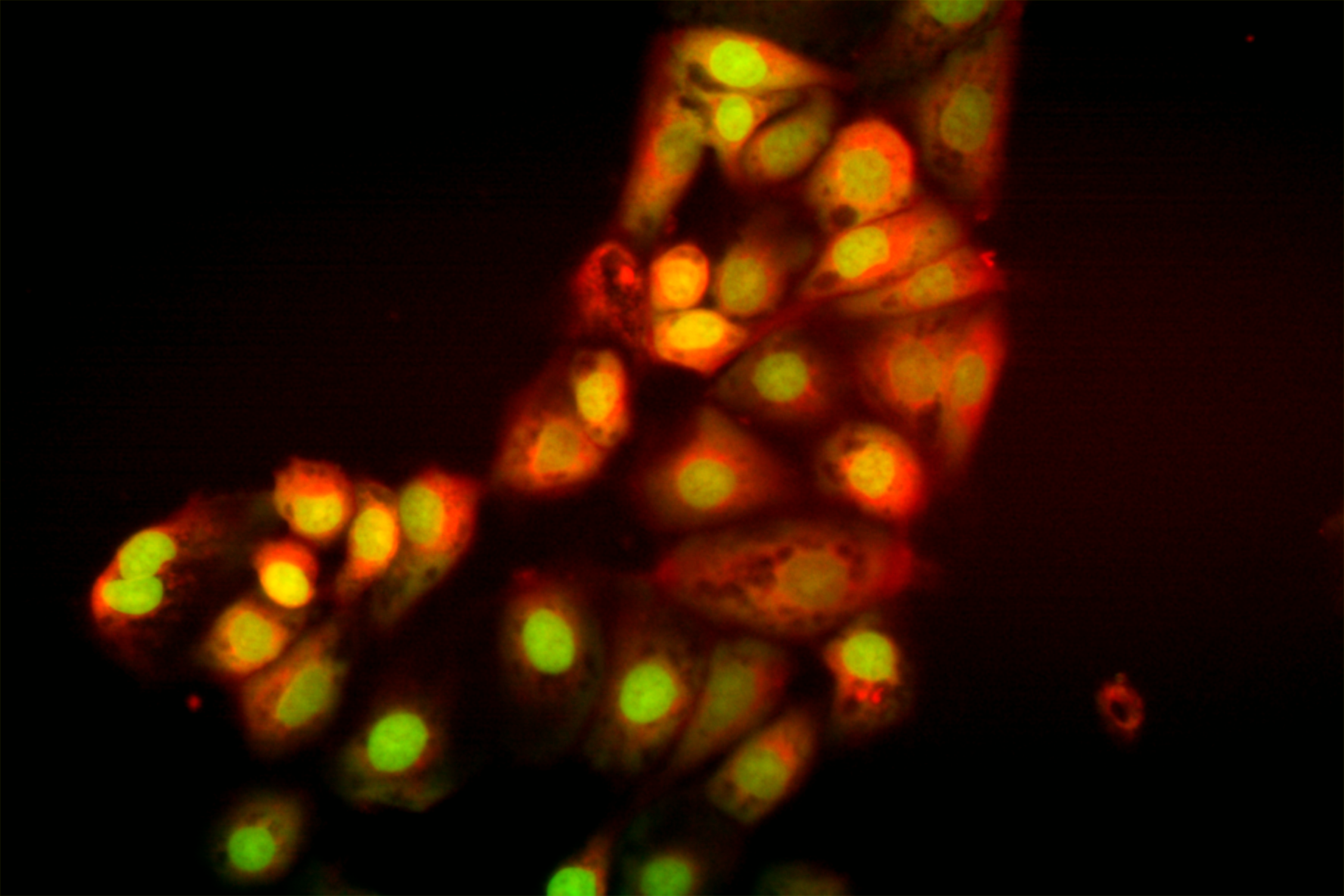

Supplement: Supplementary file 1 [file vetsci-11-00643-s001.zip › Supplementary Materials/RGB (2)(1).tif]

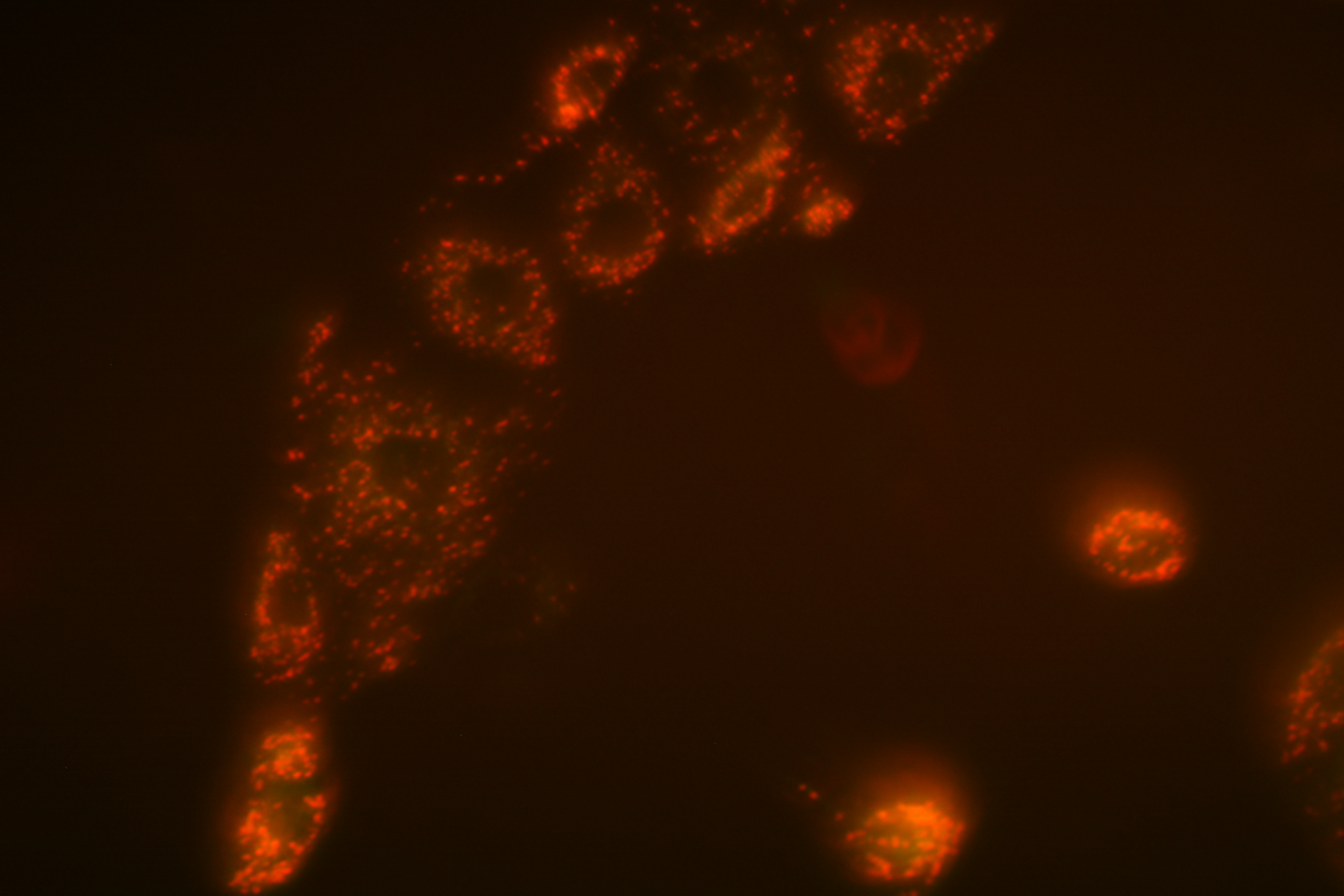

Supplement: Supplementary file 1 [file vetsci-11-00643-s001.zip › Supplementary Materials/RGB(1).tif]

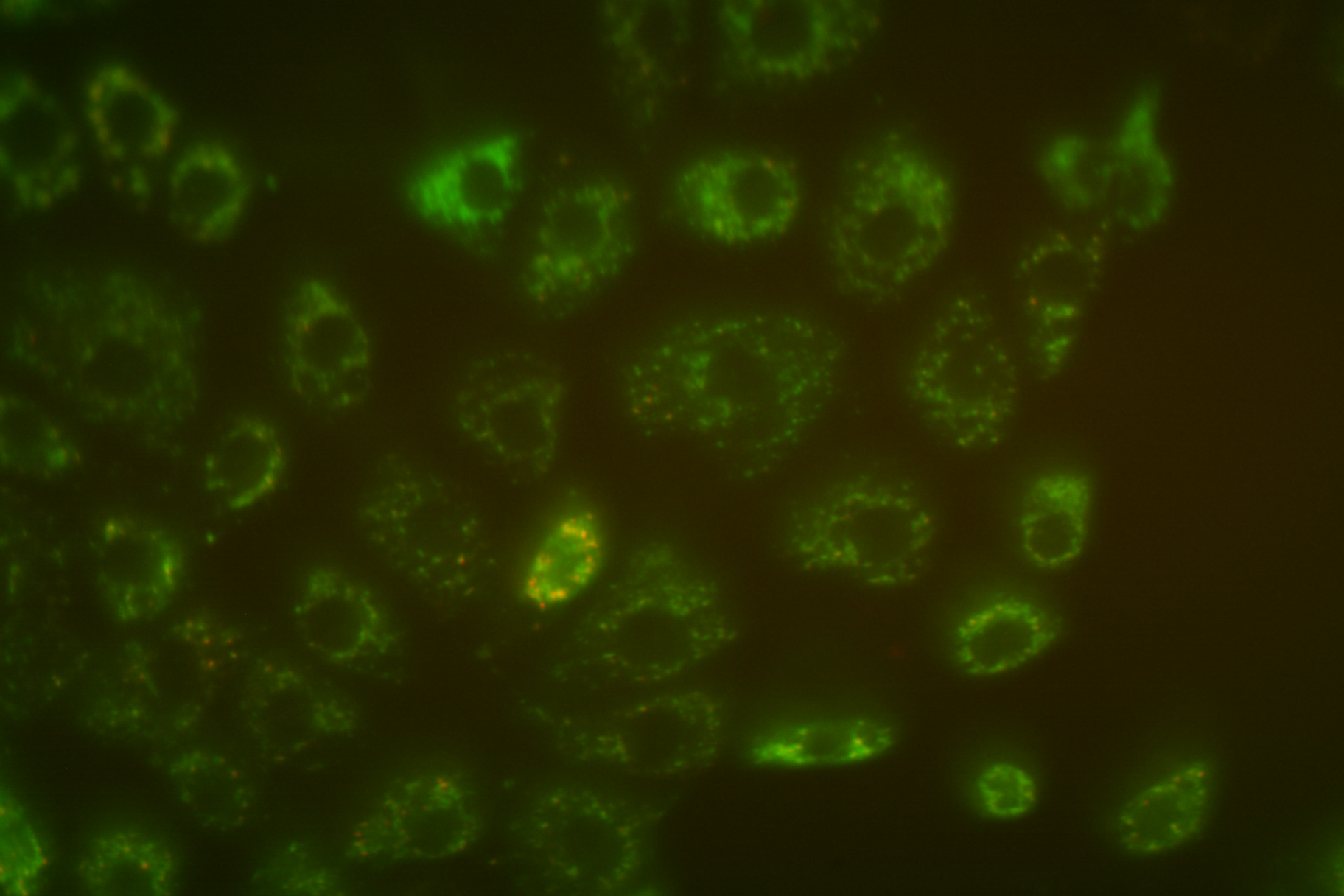

Supplement: Supplementary file 1 [file vetsci-11-00643-s001.zip › Supplementary Materials/RGB1(1).tif]

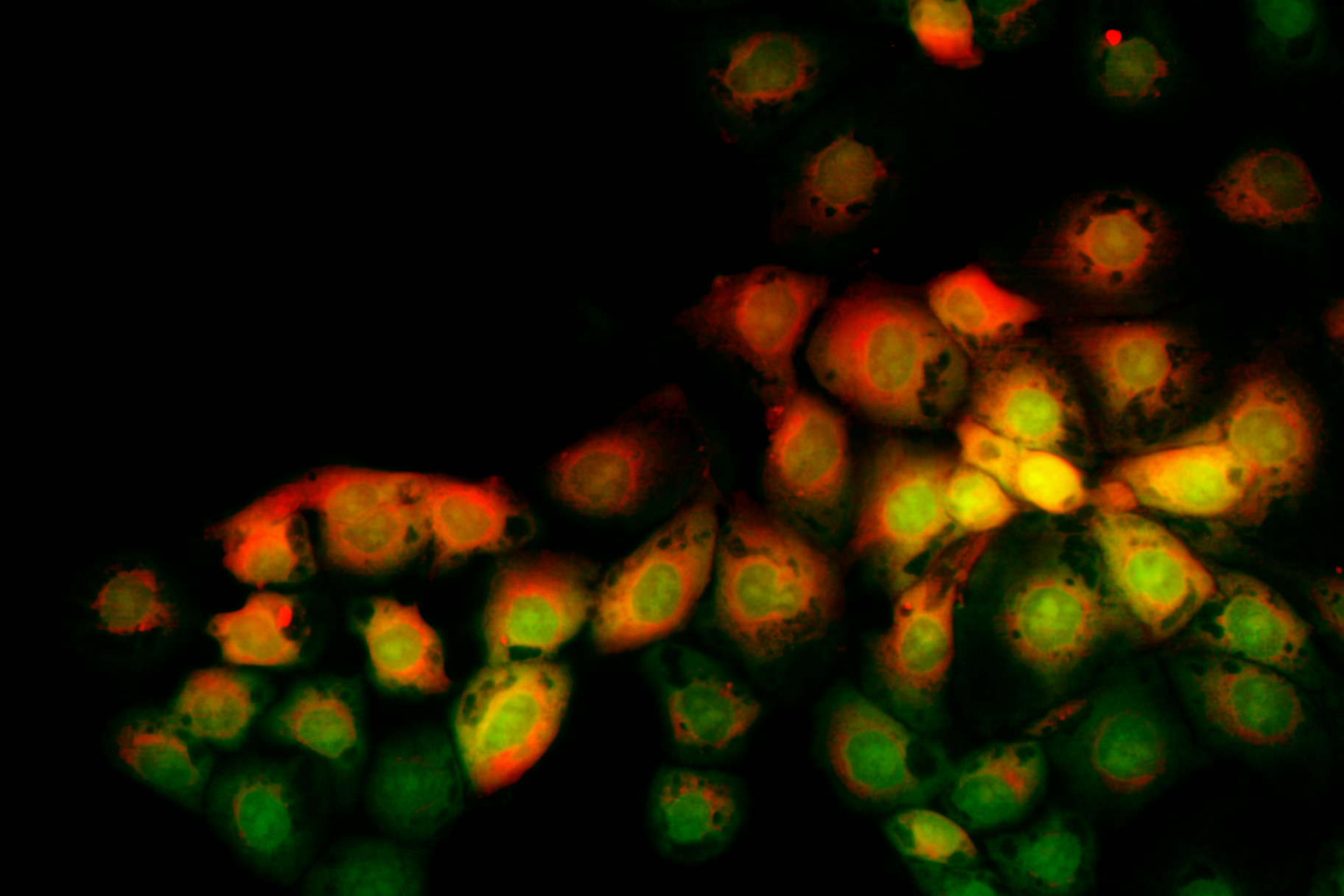

Supplement: Supplementary file 1 [file vetsci-11-00643-s001.zip › Supplementary Materials/RGBII(1).tif]
